# Two birds, one stone: hesperetin alleviates chemotherapy-induced diarrhea and potentiates tumor inhibition

**DOI:** 10.18632/oncotarget.24563

**Published:** 2018-02-23

**Authors:** Yaping Yu, Ren Kong, Huojun Cao, Zheng Yin, Jiyong Liu, Xiang Nan, Alexandria T. Phan, Tian Ding, Hong Zhao, Stephen T.C. Wong

**Affiliations:** ^1^ Department of Systems Medicine and Bioengineering, Houston Methodist Research Institute, Weill Cornell Medicine, Houston, TX, 77030, USA; ^2^ Department of Gynecology and Obstetrics, Xiangya Hospital, Central South University, Changsha, Hunan, 410008, P.R. China; ^3^ Institute of Bioinformatics and Medical Engineering, School of Electrical and Information Engineering, Jiangsu University of Technology, Changzhou, Jiangsu, 213001, P.R. China; ^4^ Iowa Institute for Oral Health Research, College of Dentistry, The University of Iowa, Iowa City, IA, 52246, USA; ^5^ Department of Pharmacy, Changhai Hospital, Shanghai, 200433, P.R. China; ^6^ Center for Biomedical Engineering, Department of Electronic Science and Technology, University of Science and Technology of China, Hefei, Anhui, 230026, P.R. China; ^7^ Cancer Treatment Centers of America at South Eastern Regional Center, Atlanta, GA, 30265, USA; ^8^ Houston Methodist Cancer Center, Houston Methodist Hospital, Houston, TX, 77030, USA

**Keywords:** chemotherapy-induced diarrhea, hesperetin, human intestinal carboxylesterase (CES2), STAT3, macrophage

## Abstract

Chemotherapy-induced diarrhea (CID), with clinical high incidence, adversely affects the efficacy of cancer treatment and patients’ quality of life. Our study demonstrates that the citrus flavonoid hesperetin (Hst) has a superior potential as a new agent to prevent and alleviate CID. In the animal model for irinotecan (CPT-11) induced CID, Hst could selectively inhibit intestinal carboxylesterase (CES2) and thus reduce the local conversion of CPT-11 to cytotoxic SN-38 which causes intestinal toxicity. Oral administration of Hst manifested an excellent anti-diarrhea efficacy, prohibiting 80% of severe and 100% of mild diarrhea in the CPT-11 administered tumor-bearing mice. In addition, a significant attenuation of intestinal inflammation contributed to the anti-diarrhea effect of Hst. Moreover, Hst was found to work synergistically with CPT-11 in tumor inhibition by suppressing the tumor's STAT3 activity and recruiting tumoricidal macrophages into the tumor microenvironment. The anti-intestinal inflammation and anti-STAT3 properties of Hst would contribute its broad benefits to the management of diarrhea caused by other chemo or targeted agents, and more importantly, enhance and reinforce the anti-tumor effects of these agents, to improve patient outcomes.

## INTRODUCTION

Chemotherapy-induced diarrhea (CID) is one of the most common and dose-limiting toxicities encountered in standard chemotherapy, targeted therapy and immunotherapy with incidence as high as 80%, an average of 4 episodes of diarrhea per patient over the treatment cycles, and more than 30% of patients experiencing severe or life-threatening situations (NCI CTC grade 3-5) [1]. Moreover, CID has been reported to last as long as ten years post-treatment [2]. Severe diarrhea is correlated with significant malnutrition and dehydration, which are linked to early death in roughly 5% patients [3]. CID, even low grades (1 or 2), significantly interferes with anti-cancer treatment [4–6], resulting in dose reductions in 22-45% of patients, dose delays in 28-71% of patients and complete treatment cessation in 3-15% of patients. In a 378 cohort retrospective study with a majority of patients experiencing grade 1 or 2 CID, 65% of patients had a reduction in chemotherapy dose intensity [6]. Several studies have demonstrated decreased overall and disease-free survival after reductions in dose intensity [7, 8]. Despite the high incidence and potential severity of CID, it is often under recognized, poorly understood and improperly managed.

Therapeutic agents commonly causing CID include chemotherapies such as irinotecan (CPT-11), 5-fluorouracil (5-FU) and capecitabine [1]; targeted therapies such as erlotinib, sorafenib and cetuximab [1], and immunotherapies such as ipilimumab, pembrolizumab and nivolumab [9]. Combinations involving chemotherapy, targeted agents and immunotherapy for cancer treatment are common and significantly increase the occurrence and severity of CID [10]. The underlying mechanisms of CID remain unclear, but are believed to result from a combination of intersecting mechanisms including inflammation, secretory dysfunction and gastrointestinal (GI) dysmotility. Current treatments for CID aim to reduce the severity of symptoms rather than combating the pathophysiological mechanisms, and often result in worsening of already existing chronic GI symptoms or triggering the onset of other side effects [11]. Identification of potential targets and development of novel treatments alleviating CID are essential to improve clinical outcomes and quality of life amongst cancer sufferers.

Most research into the mechanisms underlying CID has focused on CPT-11 and its active metabolite SN-38 [12–14]. CPT-11 is widely used for treatment of solid and liquid tumors in both children and adults. Its global consumption has experienced rapid growth since approval by US FDA in 1996 and becoming generic in 2006. It is on the WHO Model List of Essential Medicines, the most important medications needed in a basic health system. Many “next-generation irinotecan” have also been developed in recent years, such as ONIVYDE (known as MM-398, PEP02, or nal-IRI, Merrimack Pharmaceuticals, Inc.), an irinotecan-encapsulated liposomal formulation that was approved by US FDA in 2015, and others including polyethylene conjugated SN-38 (PEG-SN38, BelrosePharma Inc.), micelle nanoparticles (NK012), SN-38 conjugation with monoclonal antibody (labetuzumab-SN-38 immunoconjugates), macromolecular carrier binding CPT-11 (hyaluronic acid + CPT-11), etc.. While the “new irinotecans” have improved bioavailability and efficacy, diarrhea persists as a significant and dose-limiting adverse effect. The diarrhea is characterized by a delayed onset, significantly high incidence and lack of adequate response to conventional anti-diarrhea agents.

As a pro-drug, CPT-11 is hydrolyzed by human liver carboxylesterase (CES1) and converted to its active form, SN-38, inhibiting type I DNA topoisomerase and killing tumor cells. SN-38 undergoes further metabolism by liver UDT-glucuronosyltransferase 1A1 (UGT1A1) to the inactive SN-38G. During the enterohepatic circulation process, intestinal deletion of UGT1A1 [13] and existence of bacterial β-glucuronidase (GUS) enzyme [12] impede the detoxification of SN-38G and induce the regeneration of cytotoxic SN-38. The intestinal accumulation of SN-38 causes proliferative cell loss and inflammation in the intestinal tract, which subsequently manifests as dose dependent diarrhea.

Conventionally, the liver is considered the major organ for CPT-11 metabolism, abundantly expressing both CES1 and UGT1A1 enzymes. However, the intestinal tissue from both humans and rodents also express CES and UGT [15, 16]. More severe damage has been shown to occur in small intestine epithelium rather than in the bacterial-enriched colon after CPT-11 administration [13, 17]. Although the colon epithelial damage can be remarkably improved by GUS inhibitors, targeting GUS is insufficient to alleviate the diarrhea [17]. These results clearly indicate the existence of important intestinal factors as the mechanisms of diarrhea. As 30% of CPT-11 was found unchanged in human bile after i.v. dosing radiolabeled drug [18], and the bile duct opens into the proximal duodenum, a direct conversion of CPT-11 to SN-38 could occur within the intestine [15, 19, 20].

In the current study, we discovered that the intestinal CES2 played a key role in the CPT-11-induced intestinal toxicity. Motivated by the highly efficient translational potential of drug repositioning, we screened known drug compounds to selectively target CES2 by our previously reported strategy [17]. A group of natural flavanone compounds stood out and their IC50s and specificities were further examined *in vitro*. Among them, hesperetin (Hst) was chosen for an *in vivo* target engagement and anti-diarrhea efficacy study in mouse models. Hst had an excellent anti-diarrhea efficacy, prohibiting 80% of severe and 100% of mild diarrhea in the CPT-11 administered tumor-bearing mice. In addition to its profound inhibition of intestinal CES2 activity, a significant attenuation of intestinal inflammation also contributed to its anti-diarrhea effect. Surprisingly, we found that Hst has a synergistic anti-tumor effect when combined with CPT-11. We demonstrated that the negative regulation of STAT3 activity by Hst correlates with increased recruiting of tumoricidal macrophages into the tumor microenvironment. These novel characteristics of Hst indicate its broader benefit in the management of CID caused by other chemo or targeted agents, and more importantly, enhancing their anti-tumor effects to improve patient outcomes.

## RESULTS

### Intestinal CES2 is a potential target for CPT-11-induced intestinal toxicity

RNA-seq data from 37 normal human tissue types in The Human Protein Atlas Project (http://www.proteinatlas.org/) were analyzed. The human liver predominantly expresses CES1 with much smaller quantities of CES2, while the small intestine contains abundant CES2 with virtually no CES1 (Supplementary Figure 1A). The distinct tissue enrichment of CES2 in the small intestine may implicate a direct conversion of CPT-11 to SN-38, resulting in intestinal toxicity. CPT-11 is approved as the first-line treatment for advanced or metastatic colon cancer and gastric cancer. Neither DNA copy number nor mRNA expression of CES2 gene has any alteration between normal and cancerous colorectal or gastric tissues in the TCGA datasets (Supplementary Figure 1B-1E), suggesting that targeting CES2 may not potentiate tumor growth.

Previous studies on rats showed that CES2 inhibition decreased SN-38 in small intestine tissue and lumen, and significantly improved CPT-11 induced diarrhea; importantly, this CES2 inhibition did not affect the area under the concentration-time curve (AUC) of blood SN-38 [21]. Moreover, blocking the intestinal absorption of SN-38 in patients by using oral alkalization did not decrease the tumor response rates with the standard CPT-11 dosing [22]. These data further indicate that selectively targeting intestinal CES2 is a promising strategy to prevent CPT-11 induced diarrhea.

### Identification of selective CES2 inhibitors

Streptomycin was shown to inhibit CES2 activity and alleviate diarrhea in rats [21]. However, antibiotics are much less commonly applied in cancer patients to treat diarrhea because of many negative consequences [23]. Loperamide, an FDA approved anti-diarrhea drug, is able to specifically inhibit CES2 [24]. But it's mechanism of action is to decrease the smooth muscle motility by binding to μ-opioid receptors, thus it's not recommended to use for more than 48 hours due to paralytic ileus (FDA label). Overall, high dose loperamide improves symptoms at first occurrence of diarrhea but the incidence of grade 3-4 diarrheas remains high at 28-40% of treated patients. A number of new CES2 inhibitor compounds have been developed with distinct scaffolds [25, 26]. However, none of them have been tested on animal models due to their poor drug-like properties, thus require prolonged lead optimization and high failure rate evaluations, especially due to toxicity profiles. To increase the translational efficiency, we seek to reposition existing drugs as CES2 inhibitors. A virtual screening strategy based on CES2 structure was adopted as follows.

### Structural model of CES2

The protein structure of human CES2 is not available. However, preliminary computer modeling of the rabbit CE and human CES1 indicate that the ability of a CE to activate CPT-11 is dependent on the residues that form the entrance to the active site gorge [27]. The highly conserved structural topology of this protein family encouraged us to build a model of CES2 based on a homologue with known structure. CES1 was identified with the highest sequence similarity to CES2 in entire protein databank (https://blast.ncbi.nlm.nih.gov/Blast.cgi) [28], i.e., it shares 47% identity and 63% similarity with CES2, thus it was used as the major template to construct a CES2 model.

**Figure 1:**
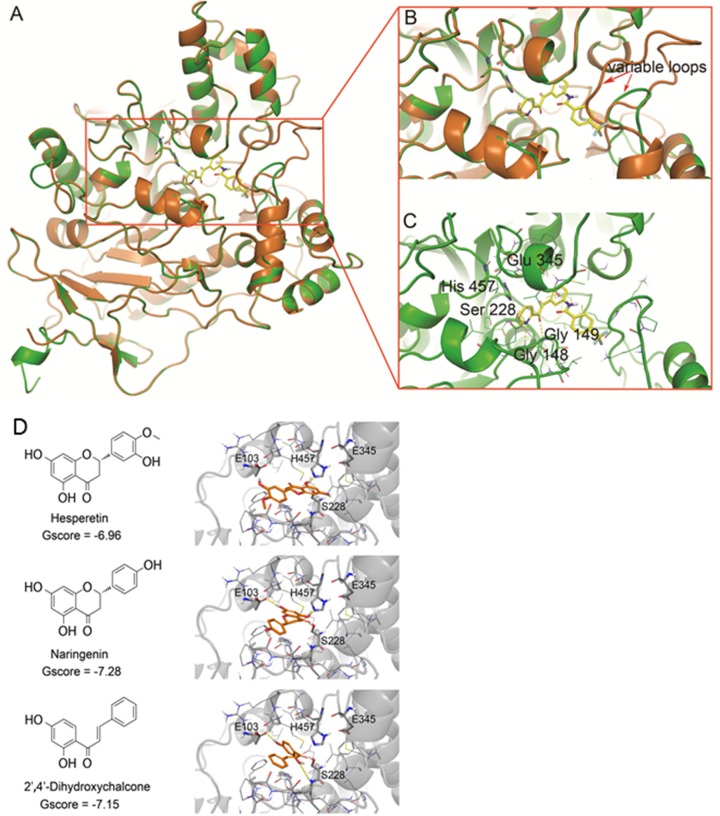
Identification of CES2 inhibitors **(A)** The homology model of human CES2 (green) was superimposed with the template structure of CES1 from 1MX1 (orange). **(B)** The enlarged image of CES2 active site. **(C)** Binding of the selective CES2 inhibitor 15f in CES2 active site. The CES2 protein was shown in the cartoon model and compound 15f was shown in stick model. **(D)** Binding poses of the identified CES2 inhibitor compounds. 2D chemical structures of hesperetin, naringenin and 2’,4’-dihydroxychalcone were shown in left panel and the corresponding docking poses were shown in right panel. In 3D models, compounds were shown in a ball-and-stick model with carbon in orange, nitrogen in blue, oxygen in red, and hydrogen in white. The CES2 protein was shown in the cartoon model and colored in grey. The residues in 5Å neighborhood of ligand binding site were represented in the line model on the left. Active site residues and the residues forming hydrogen bonds with ligand were represented in the ball-and-stick model with carbon in grey, nitrogen in blue, oxygen in red and hydrogen in white. The yellow dash lines represented hydrogen bonds between specific atoms. For clarity, all non-polar hydrogens were hidden.

Most significantly, our CES2 structural model differed from CES1 in the loop conformation adjacent to the active site (Figure 1A-1B), due to the non-conserved residues from Ser296 to Val313 (Supplementary Figure 2). This loop locates in the vicinity of the entrance of the active site and could be one determinant of substrate specificity [27]. With the exception of this loop, the CES2 model inherited most of the secondary structure from its template CES1, including the conserved topology structure of the α/β hydrolase super family. The RMSD of Cα bonds between the CES2 structural model and CES1 template was 0.77 Å. The side chain of Ser 228, Glu 345, and His 457, which form the enzyme's catalytic triad and is conserved in all human CEs, superimposed well on the template (Figure 1A-1B). The Ramachandran plot of the built model also suggested that most of the residues accommodated reasonable conformations (Supplementary Figure 3).

15f, a known CES2 inhibitor with the highest selectivity against CES1 [29], was docked to the active site of the constructed CES2 model (Figure 1B-1C). The pyridyl-ethanedione scaffold of 15f formed hydrogen bonds with the side-chain of Ser 228 from the catalytic triad, and backbone amide of Gly 148 and 149 in the active site. Its long molecular shape extended toward the variable loop of CES2 and the trifluoromethyl benzene group formed contacts with the loop surrounding residues. However, the variable loop in CES1 was much longer than that in CES2 and imposed spatial hindrance to the binding of 15f, which might be the determinant of the 400-fold difference in IC50s between CES1 and CES2 [29]. These results suggested that the homology model of CES2 is suitable to identify potent and selective CES2 inhibitors.

### In silico and *in vitro* drug screening

A total of 6,325 drug compounds from MicroSource Spectrum library, LOPAC library, Johns Hopkins Drug Library, and Prestwick library were evaluated by docking to the active site of the CES2 homology model. The compounds in these libraries are previously used drugs, withdrawn drugs, and nutrients. From the *in silico* screening, 196 compounds fulfilled the criteria of binding in the active site with a glide docking score ranked in the top 10% and hydrogen bonding with at least one of the catalytic residues S228, E345, H457, G148 or G149 (Supplementary Table 1). These compounds were then tested in the CES2 enzyme assay at one concentration of 50μM as a primary screening. Twenty-four compounds had >50% inhibition of CES2 enzyme activity, and were proceeded for confirmation on CES2 selectivity over CES1 (Supplementary Table 2). Finally, three compounds, i.e., hesperetin (Hst), naringenin and 2’, 4’-dihydroxychalcone showed selective inhibition of CES2 activity with IC50s as 2.54±0.16 μM, 9.72±0.68 μM and 1.66±0.69 μM, respectively. For Hst and 2’, 4’-dihydroxychalcone, no significant CES1 inhibition was identified even at 300μM, demonstrating >100-fold selectivity for CES2. For naringenin, a relatively lower but still considerable selectivity value (16-fold) was obtained. All the three compounds are flavonoids.

As shown in Figure 1D, all three compounds were predicted to bind within the active site of CES2 near the catalytic residues H457 and S228. The 2D structures of Hst and naringenin are very similar, differing only by substitutions on the phenol ring. Both compounds inserted the bi-aromatic ring inside the active site, locating the phenol ring on the entrance site. The direction of bi-aromatic rings varied in these compounds: Hst formed a hydrogen bond with H457, whereas naringenin formed hydrogen bonds with H457 and E103. Similar to the two compounds, the hydroxyl substituted phenol ring of 2’, 4’-dihydroxychalcone lays inside the active site and formed hydrogen bonds with E103 and S228; the other phenol ring was placed on the entrance of active site. The docking results suggest that these compounds occupy the entrance of the active site of CES2 and formed hydrogen bonds with the catalytic residues H457 and S228. Structurally, multiple hydroxyl and carbonyl moieties in flavonoids favor forming hydrogen bonds with the polar residues inside the catalytic triad [30].

### Intestinal, not liver CES activity, is inhibited by *in vivo* administration of hesperetin

In our *in silico* and *in vitro* studies, Hst was identified as a potent selective CES2 inhibitor with IC50 around 2.54 μM. Hst is a flavonoid that exists widely in plants, fruits, flowers, and foods of plant origin and exerts interesting pharmacological properties such as antioxidant, anti-inflammatory, blood lipid lowering and cholesterol lowering and is considered to contribute to health benefits in humans [31, 32].

To test the *in vivo* inhibition of CES2 activity by Hst, 20 mg/kg Hst in mice equivalent to 135 mg/60kg in human was orally administered to mice. Single oral dose of 135 mg Hst in healthy adult subjects was rapidly absorbed and the concentration in plasma observed 20 min after dosing and reached a peak in 4h. The mean peak plasma concentration (Cmax) of Hst was 2.73±1.35 μM [33], which is close to the IC50 of Hst against CES2. Microsomes of the liver and small intestinal epithelium were used to examine the CES activity, and the results showed that Hst treatment reduced the small intestinal CES activity by 56% (control=5.13±1.27 nmol/min/g protein *vs*. Hst=2.28±1.42 nmol/min/g protein, n=5, P<0.05, by Student's *t* test), while the liver CES activity did not have an obvious change (control=9.13±0.97 nmol/min/g protein *vs*. Hst=8.89±0.74 nmol/min/g protein, n=5). As the small intestine contains virtually no CES1 but abundant CES2 (Supplementary Figure 1A), these results further demonstrated that Hst inhibited *in vivo* intestinal CES2 significantly.

### Hesperetin significantly attenuates CPT-11 induced diarrhea *in vivo*

We tested the effect of Hst (20 and 100 mg/kg) on murine CT-26 tumor-bearing immuno-competent mice [17] with CPT-11 (50 mg/kg, roughly equivalent to 5 mg/kg typical human CPT-11 dose) intraperitoneally injected daily from day 1-9. Hst was given from day 1-15 and 30 min before CPT-11 administration on day 1-9. Changes of stool character were recorded twice daily to determine the severity of diarrhea. Fecal staining of skin or lose watery stool was determined as diarrhea but not severe, and only the black sticky stool (bloody diarrhea) was determined as severe diarrhea.

**Figure 2:**
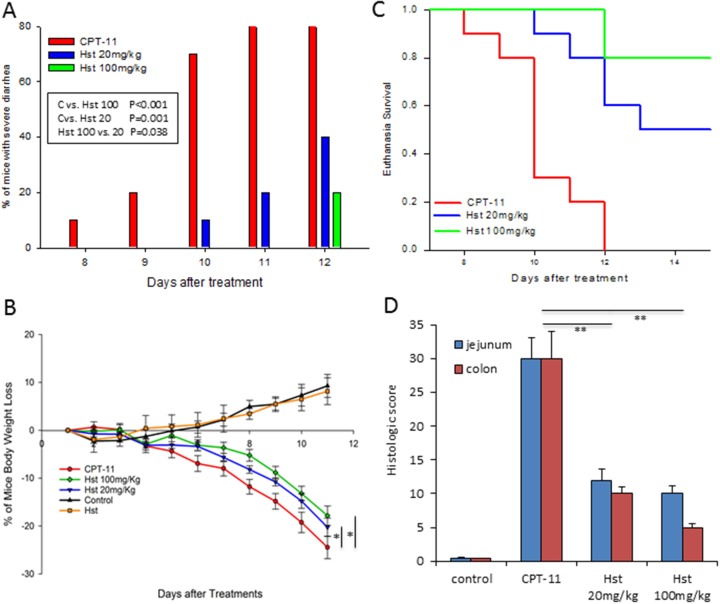
*In vivo* effects of hesperetin (Hst) against CPT-11 induced diarrhea **(A)** Hst successfully alleviated CPT-11 induced severe diarrhea. ^*^ P=0.023. **(B)** Hst protected the mice from body weight loss caused by CPT-11. ^*^ P<0.05, *vs*. CPT-11. **(C)** Hst significantly improved the euthanasia survival rate. P<0.001 for both Hst 20 and 100mg/Kg groups *vs*. CPT-11. **(D)** Tissue histology of jejunums and colons taken from mice in each group showed that Hst protected the tissue from damage. ^**^ P<0.01, *vs*. CPT-11. n=10 for each group. CPT-11: CPT-11 50mg/kg + vehicle; Hst 20mg/kg: CPT-11 + Hst 20 mg/kg; Hst 100MG/KG: CPT-11 + Hst 100 mg/kg; Hst: Hst 100 mg/kg; control: vehicle control.

Severe diarrhea was first observed on day 8 post CPT-11 treatment in the CPT-11 only group (1 out of 10 mice) and on day 11, 8 out of the 10 mice showed signs of bloody diarrhea in this group, while only 2 out of 10 mice in the Hst 20 mg/kg group were examined with severe diarrhea on day 11. Until day 12, only 2 out of 10 mice in the Hst 100 mg/kg group suffered from severe diarrhea (Figure 2A). In addition to those with severe diarrhea, 100% of mice in the CPT-11 only group developed some degree of diarrhea during the 15-day course of the entire study, but 20% and 30% of the mice in Hst 20 and 100 mg/kg groups, respectively, never showed any signs of diarrhea through the end of the study.

All mice receiving CPT-11 started losing body weight on day 4 due to the drug's cytotoxicity. During the course of the entire study, Hst significantly attenuated body weight loss of the mice in Hst 20 and 100 mg/kg groups compared with the mice in the CPT-11 only group (Figure 2B) (P<0.05 and P<0.01 respectively, by two-way ANOVA test). On day 11, the mice in the CPT-11 group had lost 25% of body weight on average, compared with 20% and 17%, respectively, in the Hst 20 and 100 mg/kg groups.

Mice in both the Hst 20 mg/kg and 100 mg/kg group had a significant improvement on the euthanasia survival (Figure 2C) (P<0.001 for both groups, by log rank test). By day 12, all mice in the CPT-11 group had to be euthanized, compared with only 40% and 20% mice in the Hst 20 mg/kg and 100 mg/kg groups. In addition, 20% of the mice in both Hst 20 and 100 mg/kg groups showed a rapid recovery in body weight 5 days after the cessation of CPT-11 (data not shown). These results indicated that Hst not only remarkably alleviated the diarrhea but also prevented the occurrence of diarrhea in some mice.

**Figure 3:**
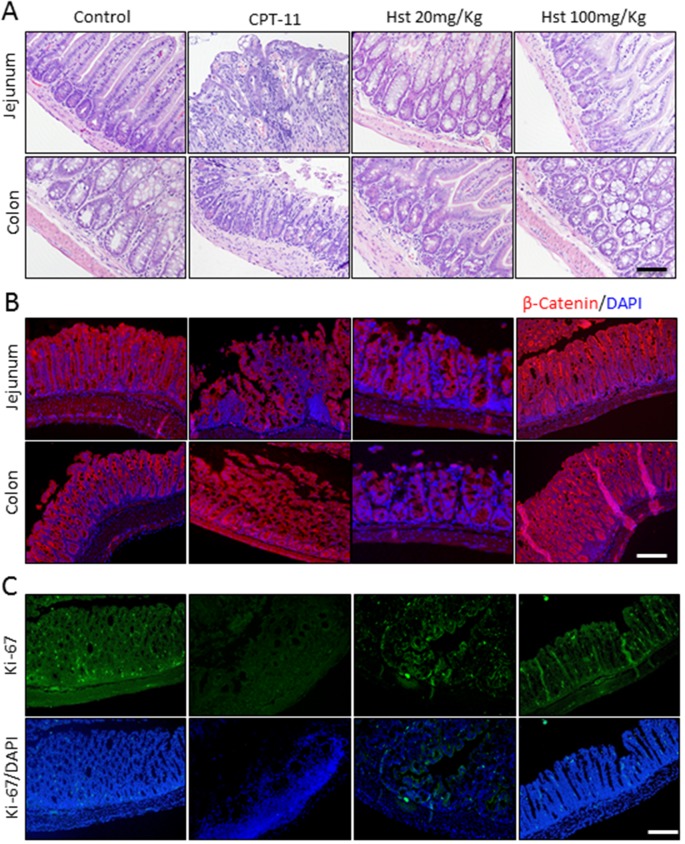
Hesperetin protects intestine and colon tissue form CPT-11 induced damage Jejunum and colon tissues from Control, CPT-11, Hst 20mg/Kg and Hst 200mg/Kg group were analyzed on day 12 respectively. Representative images of H&E staining **(A)**, immunohistological staining of β-Catenin on the integrity of membrane **(B)** and Ki-67/DAPI staining of jejunum tissue **(C)** were photographed. Scale bar.100μm.

Histopathological examination of the animal tissue indicated that oral administration of Hst protected the glandular structures of the small intestine and colon (Figure 2D). It also facilitated maintenance of Ki-67-positive proliferative cells and mucosa membrane integrity, which is damaged by CPT-11 (Figure 3A-3C). These observations further supported the *in vivo* efficacy of Hst against diarrhea.

### Hesperetin significantly attenuates intestinal inflammation

**Figure 4:**
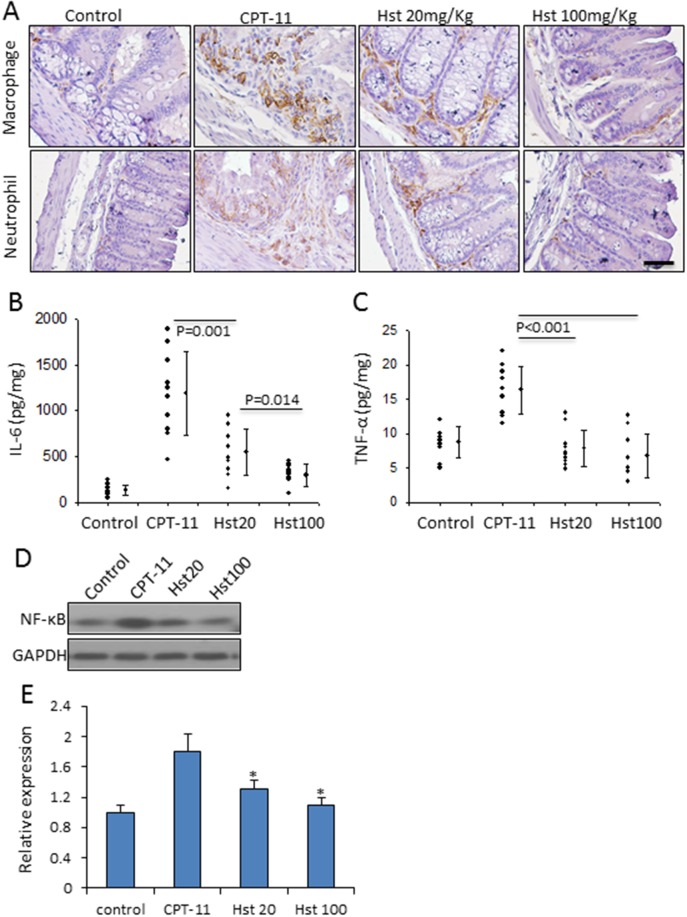
Hesperetin attenuates intestinal inflammation caused by CPT-11 **(A)** Hst inhibited the infiltration of macrophage and neutrophil in the small intestine tissue. **(B-C)** Hstsuppressed the release of IL-6 and TNF-α in the mouse plasma. **(D)** A representative western blot image showing the tissue expression of NF-κB in the mouse small intestine. **(E)** Quantification of the NF-κB western blot analysis on five samples in each group. ^*^ P<0.05, *vs*. CPT-11.

Almost all types of chemotherapeutic agents activate diverse pro-inflammatory pathways culminating in distinct histopathological changes in the small intestine and colonic mucosa [34]. In the CPT-11 only group, there were a large number of infiltrating inflammatory cells (neutrophils and macrophages) between intestinal crypt epithelium, as well as increased pro-inflammatory cytokines in mouse plasma including tumor necrosis factor-α (TNF-α) and interleukin-6 (IL-6). Hst blocked the infiltration of the aforementioned inflammatory cells and the cytokine release in a dose dependent manner (Figure 4A-4B). In addition, Hst treatment significantly down-regulated the tissue expression of NF-κB, the master regulator of innate immune responses (Figure 4D-4E). These results suggested that the anti-inflammatory effect of Hst contributes to alleviating diarrhea in the CPT-11 administered animals.

### Hesperetin synergizes with CPT-11 for tumor inhibition through negative regulation of STAT3 transcriptional activity

**Figure 5:**
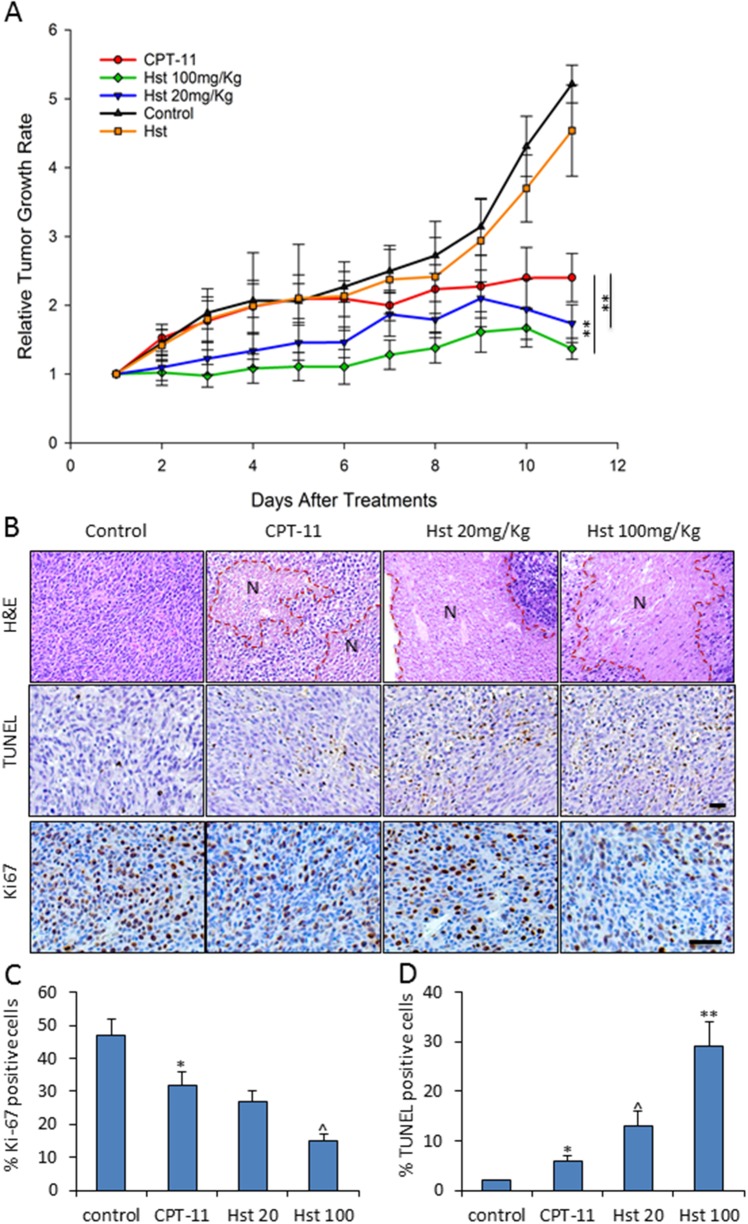
*in vivo* effects of hesperetin on tumor growth in CPT-11 treated mice **(A)** Hst enhanced the anti-tumor activity of CPT-11, while Hst only (100mg/kg) showed mild but not significant anti-tumor effect. n=10 for each group. ^**^ P<0.01, vs. CPT-11. **(B)** Representative H&E, TUNEL and Ki67 staining of the tumor samples in each treatment group. N: necrosis area. Scale bar: 20μm. **(C-D)** Quantification of the TUNEL and Ki67 immunostaining analysis on five tumor samples in each group. Each tumor was serially sectioned every 100μm, and 10 imaging fields under 20× were counted for % of positive cells. ^*^ P<0.05, vs. control; ^ P<0.05 vs. CPT-11; ^**^ P<0.01 vs. CPT-11.

Strikingly, Hst at doses of 20 and 100 mg/kg enhanced the *in vivo* antitumor activity of CPT-11 starting 5-6 days after Hst treatment (P<0.05, by two-way ANOVA test), although Hst alone only showed mild inhibition on tumor growth when given at 100 mg/kg to mice (Figure 5A). When performing the pathological examination of the mouse tumor specimens (Figure 5B), we found that all tumors receiving CPT-11 treatment showed large areas of necrosis in the H&E staining. Ki67 staining indicated less positive proliferation activity of tumor cells in the CPT-11+Hst treated mice and the high dose Hst group had a much stronger proliferation suppression effect. Reversely, TUNEL staining showed dose-dependent increases of apoptotic cells in the CPT-11+Hst treatment groups (Figure 5C-5D). *In vitro*, Hst alone did not show any potent cytotoxicity effects on different cancer cell lines including CT-26 (Supplementary Figure 4). These results indicate that the *in vivo* synergistic anti-tumor effect of Hst was not due to its direct cytotoxicity. Combined with the observation that the anti-tumor effect of Hst became obvious several days after administration, these results suggest that: 1. Hst could render an alteration on tumor transcriptional level, and 2. Hst may affect the tumor stroma to indirectly kill tumor cells.

**Figure 6:**
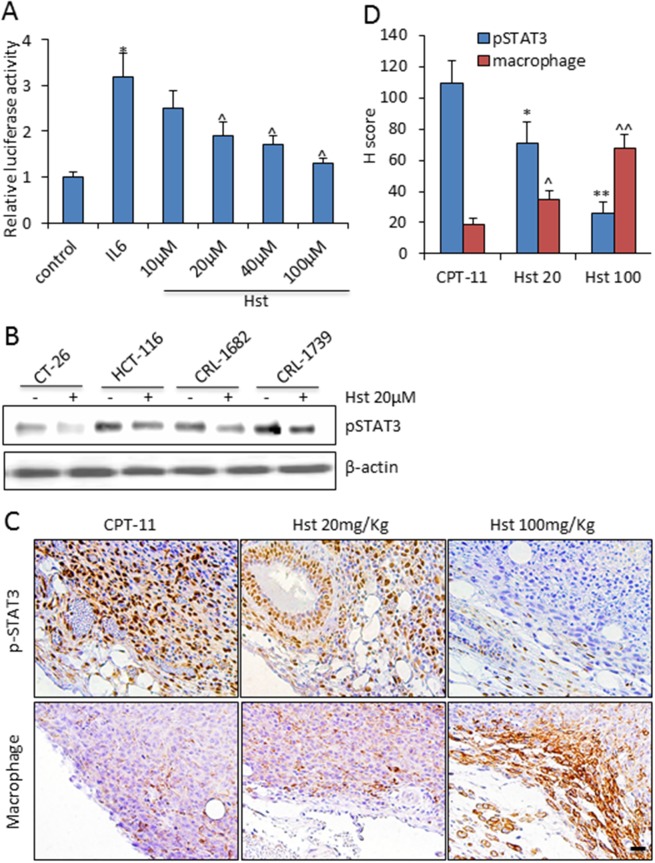
Hesperetin negatively regulates STAT3 signaling **(A)** *In vitro* effect of Hst on STAT3 activity was measured using STAT3 Cignal Reporter Assay on HEK293 cells. Experiments were done in triplicates, and the standard deviation is indicated. ^*^ P<0.05 vs. control; ^ P<0.05 vs. IL-6. **(B)** Western blot analysis of pSTAT3(Tyr705) in indicated cancer cell lines upon IL-6 100ng/ml stimulation for 90 min. **(C)** Representative immunohistochemistry staining of pSTAT3 and F4/80 (macrophage) in tumor samples from each group. Scale bar: 20μm. **(D)** Quantification of the immunohistochemistry staining of pSTAT3 and F4/80+ macrophage in five tumor samples from each group. Each tumor was serially sectioned every 100μm, and 10 imaging fields under 20× were counted for the number of positive cells. H score was calculated by multiplying the fraction of positively stained tumor (percentage) by staining intensity (0, 1+, 2+, or 3+). Intensity of immunoreactivity was scored (0 and 1+ indicates negative; 2+, indeterminate; and 3+, positive for overexpression), and the percentage of tumor staining positive was visually estimated by pathologists. ^*^ or ^, P<0.05, vs. CPT-11; ^**^ or ^^, P<0.01 vs. CPT-11.

To further investigate the tumor inhibition mechanism of Hst, we performed a transcriptional drug signature analysis by using the Library of Integrated Network-based Cellular Signatures (LINCS) database (http://lincs.hms.harvard.edu). We ranked 16,249 drugs in the LINCS database based on their treatment transcriptional similarity on cell lines with the glycoside Hst (Supplementary Figure 5). The #1 top ranked similar drug is withaferin-A (P<0.00001, by Kolmogorov-Smirnov test), a potential STAT3 inhibitor [35]. STAT3-inhibitor-VI [36] was also in the top 1.5% of ranked drugs (P<0.01, by Kolmogorov-Smirnov test). Ingenuity pathway analysis (IPA) of the drug signatures from the LINCS database further revealed that glycoside Hst could significantly down-regulate *JAK2/STAT3 signaling* (P=6.24×10^-30^) and *IL-6 induced STAT3 signaling* (P=6.81×10^-16^), which is similar to the pathways of withaferin-A and STAT3-inhibitor VI (Supplementary Figure 6), indicating that Hst is a potential STAT3 inhibitor. In cell culture, Hst was shown to inhibit IL-6 induced STAT3 reporter activity at 20-100μM (Figure 6A). *In vitro* treatment with Hst on various cancer cell lines showed significant down-regulation of IL-6 induced p-STAT3 (Tyr705) expression (Figure 6B). In the mouse tumors, high levels of nuclear p-STAT3 were observed at the tumor edge in association with both tumor cells and stromal cells (Figure 6C). Hst treatment remarkably suppressed the p-STAT3 expression in the tumors (Figure 6D). In the molecular docking study, Hst was shown to interfere the binding of phosphorylated peptide and the dimerization of STAT3 (Supplementary Figure 7).

STAT3 plays a crucial role in tumor immunosuppression, which enables tumor to evade immune surveillance [37]. Because of the particular abundance of macrophages at the tumor edge, we found that in the Hst-treated immunocompetent mice, suppressed expressions of p-STAT3 were correlated with a significant increase in macrophage infiltration at the tumor edge (R^2^=0.97) (Figure 6D). Emerging data supports that peritumoral macrophages are tumoricidal, although intra-tumoral macrophages are considered to be pro-tumoral [38]. Taken together, our results demonstrate that the negative regulation of STAT3 activity by Hst imposed a tumor inhibition synergy with CPT-11 through recruitment of tumoricidal macrophages into the tumor microenvironment.

## DISCUSSION

We report here the novel finding that the citrus flavonoid hesperetin has a superior potential to be a new agent to prevent and alleviate chemotherapy-induced diarrhea. Hesperetin could selectively inhibit intestinal CES2 and thus reduce the local conversion of CPT-11 to SN-38 in causing intestinal toxicity. Moreover, hesperetin was found to impose a tumor inhibition synergy with CPT-11 through suppressing the STAT3 activity and recruiting the tumoricidal macrophages into the microenvironment.

The mechanism of Hst against CPT-11-induced diarrhea is delineated in two aspects. First, Hst protects intestines from the initial damage caused by CPT-11, as it eliminates the local conversion and accumulation of cytotoxic SN-38. To this end, Hst performs a preventive role. Second, Hst suppressed the local intestinal inflammation. Inflammation is the fundamental pathophysiological mechanism of all CIDs. Once activated by chemotherapy, NF-κB acts to induce gene expression and production of pro-inflammatory cytokines, which in turn lead to tissue injury and apoptosis. The secreted cytokines can also stimulate secretion, in effect imposing a secretory component on top of an inflammatory diarrhea. Reactive oxygen species (ROS) from inflammatory cells can damage or kill intestinal epithelial cells, which are replaced with immature cells that typically are deficient in the brush border enzymes and transporters necessary for absorption of nutrients and water. In this way, components of an osmotic (malabsorption) diarrhea are added to the problem. In our study, Hst was shown to significantly attenuate intestinal inflammation, i.e., blocks the infiltration of inflammatory cells and the cytokine release, as well as down-regulates the tissue expression of NF-κB. Hst has been previously reported as an anti-inflammation and anti-oxidant agent [32]. We thus conclude that the outstanding anti-diarrhea efficacy of Hst in the CPT-11 administered animals is attributed to its anti-CES2 activity combined with its anti-inflammatory activities. To this end, we envision that the anti-inflammatory effect of Hst may also benefit the management of CID caused by other chemo or targeted agents. The same concept of using Budesonide (a corticosteroid medication) and Celecoxib (a nonsteroidal anti-inflammatory drug) to alleviate CID has been reported with good efficacy [1].

In addition, Hst is superior to conventional anti-diarrhea agents because of its ability on negative regulation of STAT3 transcriptional activity. Studies have shown that activation of STAT3 pathways lead to the transcription of target genes necessary for cellular proliferation [39], and aberrant STAT3 activation has been found in many solid malignancies, including colorectal cancer [40, 41]. Furthermore, STAT3 is generally accepted as a target for inducing apoptosis in solid and hematological tumors [42]. Although CES2 has been shown to express within tumor tissue [43], our data demonstrate that Hst can synergize the tumor inhibition of CPT-11 through negative regulation on tumor STAT3 activity. In the animal study, inhibition of tumor cell proliferation and induction of apoptosis were enhanced by adding Hst to the CPT-11 regimen. More interestingly, a significant recruiting of macrophages was present at the local tumor microenvironment, especially at the tumor margin. Accumulating data indicate that peritumoral macrophages are likely to have less exposure to tumor-derived cytokines and are located in less hypoxic areas, thereby they differentiate into a tumoricidal rather than pro-tumoral phenotype [38]. Significant STAT3 activation was also observed in the tumor stroma in our study. STAT3 activity promotes the production of immunosuppressive factors that activate STAT3 in diverse immune-cell subsets, altering gene-expression programs and, thereby, restraining anti-tumor immune responses [44]. In our study, the cytotoxic agent CPT-11 induced large areas of necrosis in the mouse tumors, and the dead cell fragments are immunogenic to evoke anti-tumor effects. Thus, Hst, by suppressing the stromal STAT3 activity would also contribute to the tumor inhibition synergy with CPT-11.

As noted above, many other therapeutic agents used alone or in combination commonly cause diarrhea [1, 9]. Also, accumulating evidence indicates that overcoming tumor immunosuppression makes a critical contribution to enhancing anti-tumor efficacy. From these points, further studies of hesperetin are warranted to explore whether the anti-intestinal inflammatory effect alone would be enough for achieving an anti-diarrhea efficacy in other CID models, and whether the anti-STAT3 effect contributes to anti-immunosuppression that synergizes with other therapeutic agents for tumor inhibition.

Hesperetin has been empirically proven to have no side-effects since historically humankind has been ingesting citrus fruits for a long time. However, we note that CES2 is also thought to be responsible for the hydrolysis of other xenobiotics [45]. In some instances, CES2 may contribute to converting inactive prodrugs to their active metabolites, similar to the conversion of CPT-11, including capecitabine, the antibiotics Ceftin and Vantin. However, more commonly, CES2 may contribute to hydrolyzing esterified drugs to inactive products that are then excreted, such as flestolol, meperidine, lidocaine and cocaine. Therefore, co-administration of Hst may alter the half-life of these drugs. Attention should always be taken when using Hst in patients given esterified drugs at the same time.

## MATERIALS AND METHODS

### Homology modeling of CES2

The sequence of *Homo sapiens* CES2 was retrieved from the Uniprot database with accession number O00748. NCBI Blast was used to search the protein structure database with CES2 sequence as query. There was about 47% identity and 63% similarity between the sequences of CES2 and CES1. The crystal structure of CES1 (PDB code 1MX1) was selected as the major template with relatively high resolution of 2.4 Å [46]. However, the sequence segment between residues S296-V313 is not conserved between CES1 and CES2 (the numbering of CES2 amino acid residues is based on the canonical sequence of O00748). The corresponding sequence of extracellular cholinesterase-like domain of the synaptic protein neuroligin 4 (PDB code: 3BE8) showed relatively higher similarity in this specific area and was chosen as the secondary template for CES2 model building [47]. Prime in Maestro (www.schrodinger.com) was used to do the multi-template homology model building with default settings. The resulting structural model was submitted to a two-step energy minimization by using MicroModel in Maestro. The loop regions were minimized with constraints on the other regions and then the whole structure was minimized without constraints to discard the high-energy interactions. Tacrine from 1MX1 was kept during the minimization process to maintain the binding site geometry. Ramachandran plot tool in Maestro was used to evaluate the quality of final model.

### In silico screening protocol

The docking software Glide from Schrodinger.com was used for *in silico* screening due to its good enrichment performance in order to rank the active molecules in the top of the dataset with decoys. The CES2 structure model built by homology modeling was used as receptor. The grid box center was set according to position of tacrine with 20Å×20Å×20Å in dimension to include the residues of the entire catalytic cavity. Chemical structures from MicroSource Spectrum, LOPAC, Johns Hopkins Drug library, and Prestwick library were processed by LigPrep in Schrodinger to assign the protonation states under physiological conditions, to enumerate stereoisomers and tautomers, and to generate energetically favorable 3D conformations [48]. The standard precision (SP) parameter set was used for the docking experiments with default parameter values. Several criteria were taken into consideration in drug selection: 1) ranking in the top 10% according to Glide score; 2) binding in the neighborhood of the active site; and 3) hydrogen bonding with at least one of the catalytic residues Ser 228, Glu 345, His 457, Gly 148 or Gly 149. The potential CES2 inhibitors were chosen to determine the activity and selectivity by enzyme-based CES1/CES2 assays.

### *In vitro* CES1/CES2 enzyme-based assay

The recombinant purified CES2 and CES1 enzymes, and 4-NPA were purchased from Sigma-Aldrich with product IDs E0412, E0162, and N8130, respectively. Hesperetin, naringenin, and 2’, 4’-dihydroxychalcone were purchased form Fisher Scientific (>95% purity). All compounds were dissolved in DMSO at 10mM. In the screening assay, the compounds were diluted in 50mM HEPES and the final compound concentration in the reaction system was adjusted to 50μM. In the IC50 determination assay, selected compounds were diluted in 50mM HEPES to obtain ten final concentrations from 300μM to 0.005μM with 3 fold decrement. The assays were conducted at 300μl total volume in 96-well plates. Reactions consisted of the following: 100μL 50mM HEPES with 20 units of enzyme, 10μL compound solution (various concentrations), 2μL 300mM 4-NAP, and 188μL 50mM HEPES, pH 7.4. The compound and 4-NAP solution were added into the plate first and then the reaction was initiated by the addition of enzyme solution. After 15 minutes incubation at 25°C, absorbance was measured using a microplate reader at 405nm in FLUOstar Omega Microplate Reader. The CES1/CES2 inhibitor compound bis-(4-nitrophenyl) phosphate (BNPP) was used as positive control in all the assays.

### *In vivo* experiments

Irinotecan hydrochloride (I1406) was purchased form Sigma-Aldrich (>99% HPLC purified grade). CPT-11 was dissolved in ddH_2_O to make a stock of 20mg/mL and stored at room temperature for a maximum of 2 hours prior to use. Hesperetin was dissolved in carboxymethyl cellulose solution (CMC). Healthy 6-8 week old female Balb/cJ mice (000651) were purchased from Jackson Laboratories, Bar Harbor, ME. Mice were housed in conventional metabolic cages (N=1/cage). All studies were conducted in accordance with the guidelines of the Animal Care and Use Committee of Houston Methodist Research Institute.

CT-26 cell line was used to make the tumor bearing mouse model by s.c. injection of 0.02mL cells in PBS at 5×10^7^ cells/mL into the posterior mid-dorsum. Tumor volumes were estimated by the formula π/6×a^2^×b, where a is the short and b is the long axis. When tumor volume reached roughly 100mm^3^ (~10 days after implantation, defined as day 1), mice were randomly separated into 5 groups, *1). CPT-11 only group*, receiving CPT-11 50mg/kg + vehicle *2). CPT-11+Hst 20mg/kg*, *3). CPT-11+Hst 100mg/kg*, *4). Hst 100mg/kg only*, and 5) *vehicle control group*. CPT-11 was administrated by i.p. injection from day 1 to day 9 while Hst were administrated by oral gavage from day 1 to the end of study. An equivalent dosage of saline or CMC were administrated by i.p injection and oral gavage as vehicle, respectively. Mice were examined daily for signs of diarrhea (fecal staining of skin, lose watery stool) and bloody diarrhea (black sticky stool), as well as tumor growth. After sacrifice, jejunum, ileum and colon samples were dissected. Tissues were formalin-fixed and paraffin embedded for histological examination using 4μm-thick, 100μm step serial sections stained with H&E. A histologic score to evaluate inflammation, epithelial changes and mucosa architecture for each slide was calculated as described previously [17, 49]. For immunohistochemical staining, sections were stained with antibodies against Ki67, β-catenin, F4/80, Ly-6B.2 and pSTAT3 (Tyr705) overnight at 4°C. Slides were then washed and stained with the appropriate secondary antibodies. Mounted slides were examined under Olympus BX61 upright microscopy (HMRI Advanced Cellular and Tissue Microscope Core Facility).

### *Ex vivo* liver and small intestinal CES assay

Microsomes of the liver and small intestinal epithelium were prepared from animals with or without Hst treatment. Animals were killed by exsanguination at 4h after single oral dosing of Hst. Their liver and small intestines were removed immediately and cooled in cold physiological saline. Small intestine epithelium was scraped from the intestine with slide glass. Tissues were homogenized in 1.15% KCl on ice by a Teflon homogenizer, followed by centrifugation at 9,000×g for 10min at 4°C. The supernatant was re-centrifuged at 105,000×g for 60 min at 4°C, resulting in sedimentation of the microsome. The microsomal pellet was re-suspended in 1.15% KCl. To measure CES activity, microsomes (protein content: 1 mg/mL) were incubated with 4-NAP solution in phosphate-buffered saline at 37°C, and 50μL aliquots were sampled up to 20 min.

### LINCS drug similarity analysis

Whole genome expression signatures for ~16,000 single drug treatment were downloaded from Broad LINCS/CMAP project (www.lincscloud.org). To find small molecules that have similar transcriptional profiles as Hst, we used K-S statistics to compute similarity scores for all small molecules in LINCS data [50]. LINCS only contains drug signatures and genotype data for hesperidin, the pro-drug of Hst that is 99% metabolized in cells to generate Hst, which was used as the substitute of Hst in the analysis. For each one of the 18 expression signatures for hesperidin treatment, K-S statistics was used to prioritize all signatures for other compound treatments on the same cell line according to their similarity to hesperidin treatment. For each compound, the lists of top 100 up-regulated and down-regulated genes were extracted from the expression signature, and the similarity of each compound's signature with that of hesperidin treatments was defined using K-S statistics based on the overlap between such lists of top ranked up- and down- regulated genes. We then consolidated the KS scores corresponding to 18 hesperidin signatures and generated an individual score denoting each compound's similarity with hesperidin. The expression data were permuted 1,000 times, and K-S statistics were used to calculate a significance score for each compound regarding the expression level similarity to hesperidin.

### Cell culture

CT-26 (murine colon cancer), HCT-116 (human colon cancer), CRL-1739 (human stomach gastric cancer) and CRL-1682 (human pancreatic cancer) cell lines were purchased form ATCC. All cells were cultured in T-75 flasks (Falcon). All media were purchased from Life Technologies and supplemented with 10% fetal bovine serum (Sigma) and 1% penicillin-streptomycin (Fisher Scientific). CT-26 and CRL-1682 were cultured in RPMI-1640 medium. CRL-1739 and HCT-116 were cultured in DMEM medium. All cells were cultured in a 37° C, 5% CO2 incubator.

### STAT3 reporter activity assay

STAT3 transcription factor activity was measured using the Cignal STAT3 Reporter Assay Kit (Qiagen) according to the manufacturers’ instructions. HEK293 cells were transfected with the Renilla luciferase reporter plasmid. Twenty-four hours later, cells were treated with Hst for 24h, and were incubated with 100 ng/ml IL-6 for 90 min before lysed. Subsequently, Renilla luciferase activity was measured in a microplate reader. Relative transcription factor activity was calculated by dividing relative light units (RLU) of the STAT3-specific reporter and the negative control reporter.

### Western blot analysis

RIPA buffer was purchased form Fisher Scientific. Xpert protease inhibitor cocktail solution 100X and Xpert phosphatase inhibitor cocktail solution 100X were purchased from GenDEPOT. 4–20% Mini-PROTEAN® TGX^™^ Gel and Trans-Blot® Turbo^™^ Mini Nitrocellulose Transfer Packs were purchased form Bio-Rad. Rabbit NF-κB and phospho-Stat3 (Tyr705) primary antibody, mouse β-Actin (8H10D10) primary antibody, and anti-mouse IgG HRP conjugated, anti-rabbit IgG HRP conjugated secondary antibody were purchased from Cell Signaling Technology.

### Statistical analysis

Data are expressed as means ± SD. To compare groups, we used the Student's two-tailed t test or the Mann-Whitney rank sum test. To compare frequencies of severe diarrhea, we used Holm-Sidak test with days and groups as two factors. To compare euthanasia survival, we used log-rank test. To compare tumor growth, we used Two-Way ANOVA test with days and groups as two factors. To assess correlation, we calculated the Spearman's rank correlation coefficient. P<0.05 was regarded as statistically significant. We performed all calculations with SigmaPlot statistical software (version 11.2; Systat Software Inc. Chicago, IL).

## SUPPLEMENTARY MATERIALS FIGURES AND TABLES






