# Supplementary material for: Two birds, one stone: hesperetin alleviates chemotherapy-induced diarrhea and potentiates tumor inhibition
Source: Oncotarget. 2018 Feb 23;9(46):27958–73. doi: 10.18632/oncotarget.24563 (PMC6021345; doi:10.18632/oncotarget.24563)
Supplement: Supplementary file 1 [file oncotarget-09-27958-s001.pdf]

Two birds, one stone: hesperetin alleviates chemotherapy-induced diarrhea and potentiates tumor inhibition

SUPPLEMENTARY MATERIALS

Molecular docking study of Hst with STAT3

Methods: One Monomer of SH2 domain of STAT3 were extracted from the crystal structure (PDB code 1BG1 [1]). Protein Preparation Wizard workflow in Maestro 9.2 was used to assign bond orders assigned and add hydrogen atoms. All hydrogen atoms were minimized to reach the convergence of RMSD =0.3 Å with OPLS force field. A grid-enclosing box was centered on the GLU 638 to enclose residues located within 20 Å, where the phosphorylated peptide located. A scaling factor of 1.0 was set to van der Waals (VDW) radii of those receptor atoms with partial atomic charge less than 0.25. Hst was docked to STAT3 by using Glide with SP (Standard Precision) parameter set.

Results: Supplementary Figure 7 showed the docking pose of Hst and its relative position when compared to the phosphorylated peptide. The hydroxyl group from the bi-aromatic ring formed hydrogen bonds with LYS626 and GLN635. And the hydroxyl from the phenol ring hydrogen bonded with GLU638. Although Hst didn't locate inside the phosphorylated TYR pocket, it formed spatial hindrance with other residues from the phosphorylated peptide, such as LEU706, LYS707. Thus it may interfere the binding of phosphorylated peptide and the dimerization of STAT3.

REFERENCE

1. Becker S, Groner B, Müller CW. Three-dimensional structure of the Stat3beta homodimer bound to DNA. Nature. 1998; 394:145–51.

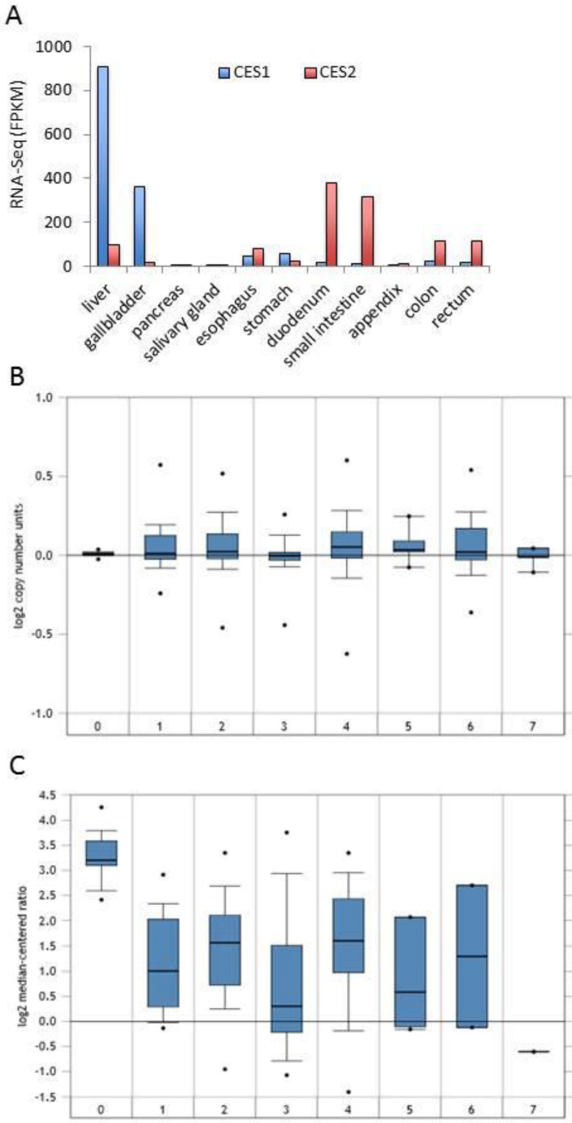

Supplementary Figure 1: Organ distribution and gene expression of CES2 in normal and cancer tissues. (A) Distribution of CES1 and CES2 mRNA in normal human gastrointestinal system. **(B)** CES2 Copy Number in TCGA Colorectal 2 with 1,172 samples. 0. normal control (581); 1. Cecum Adenocarcinoma (88); 2. Colon Adenocarcinoma (284); 3. Colon Mucinous Adenocarcinoma (55); 4. Rectal Adenocarcinoma (105); 5. Rectal Mucinous Adenocarcinoma (8); 6. Rectosigmoid Adenocarcinoma (46); 7. Rectosigmoid Mucinous Adenocarcinoma (5). **(C)** CES2 mRNA Expression in TCGA Colorectal with 237 samples. 0. normal control (22); 1. Cecum Adenocarcinoma (24); 2. Colon Adenocarcinoma (102); 3. Colon Mucinous Adenocarcinoma (20); 4. Rectal Adenocarcinoma (60); 5. Rectal Mucinous Adenocarcinoma (6); 6. Rectosigmoid Adenocarcinoma (2); 7. Rectosigmoid Mucinous Adenocarcinoma (1).

(*Continued*)

D

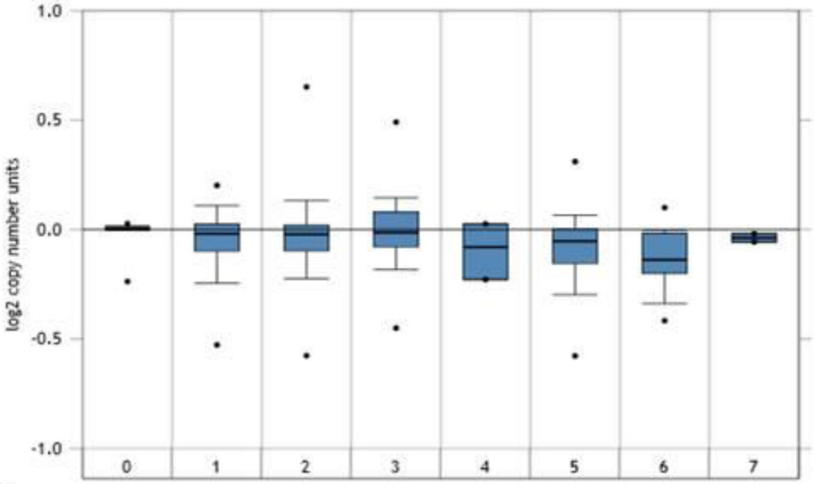

E

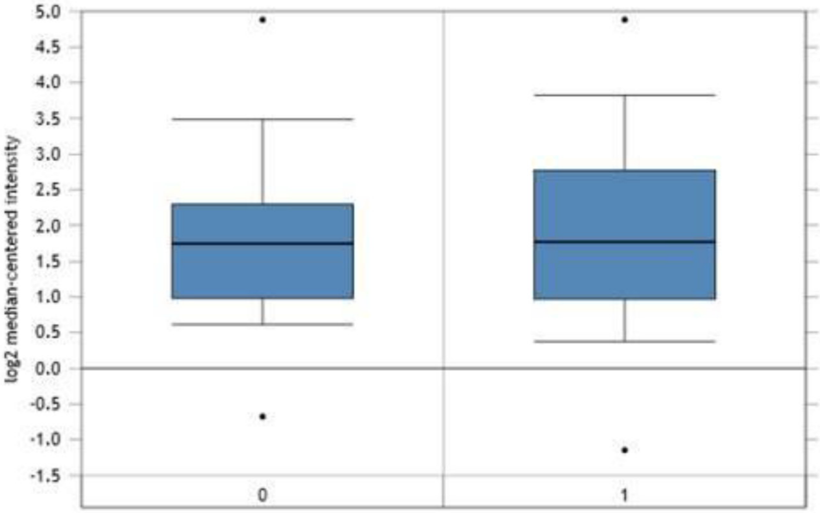

Supplementary Figure 1 (*Continued*): (D) CES2 Copy Number in TCGA Gastric with 637 samples. 0. Normal control (330); 1. Diffuse Gastric Adenocarcinoma (45); 2. Gastric Adenocarcinoma (173); 3. Gastric Intestinal Type Adenocarcinoma (41); 4. Gastric Papillary Adenocarcinoma (3); 5. Gastric Tubular Adenocarcinoma (31); 6. Mucinous Gastric Adenocarcinoma (12); 7. Signet Ring Cell Gastric Adenocarcinoma (2). **(E)** CES2 mRNA Expression in Cui Gastric with 160 samples (Nucleic Acids Res. 2011;39(4):1197-207). 0. Normal control (80); 1. Gastric Cancer (80).

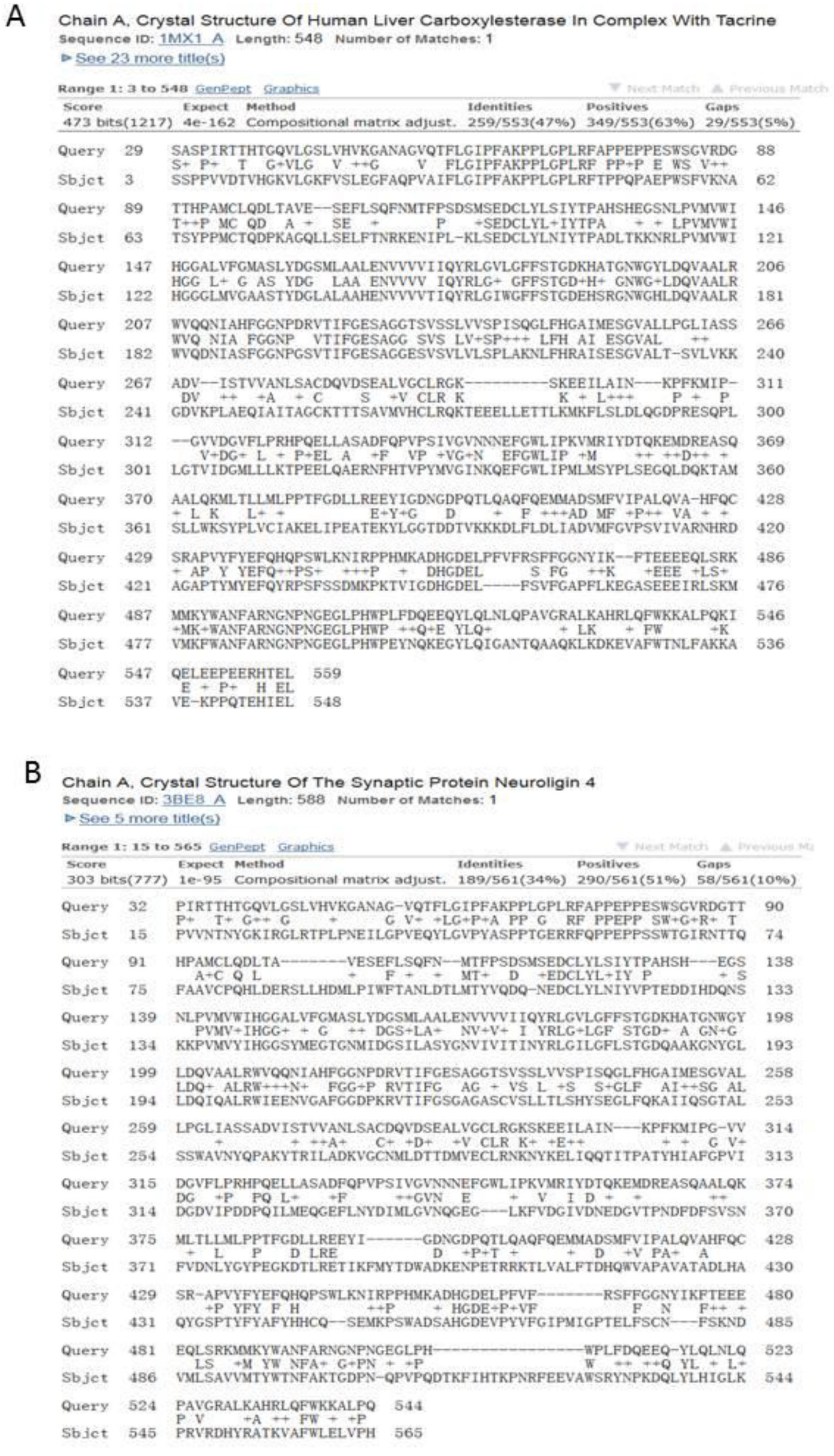

Supplementary Figure 2: Sequence alignment of CES2 with 1MX1 **(A)** and 3BE8 **(B)**. For sequence S296-V313, there are more than 10 gaps when align with 1MX1. Apparently, CES2 shares higher sequential similarity with 3BE8 in this area.

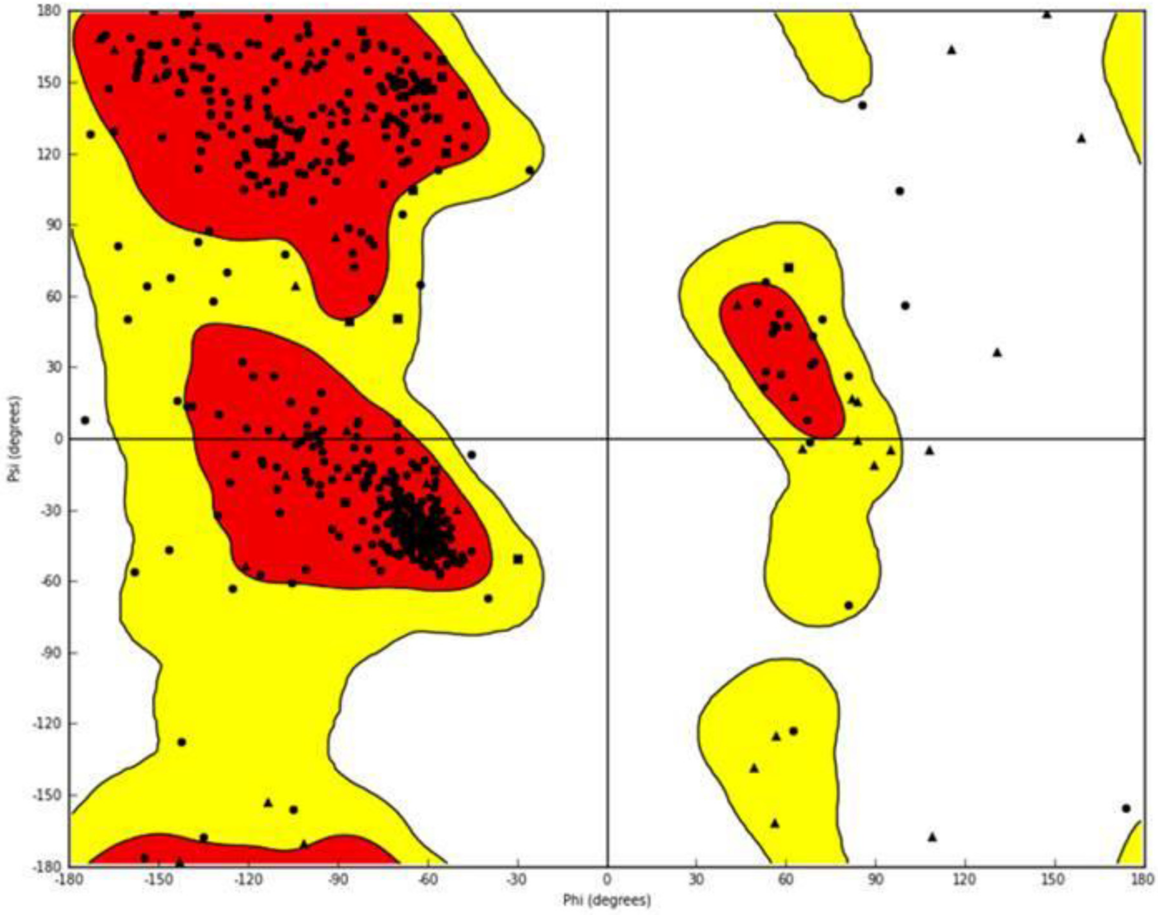

Supplementary Figure 3: Ramachandran plot mapping of the built model. Most of the residues located in the reliable (red) or acceptable (yellow) area, indicating the reliable of the built homology structure of CES2.

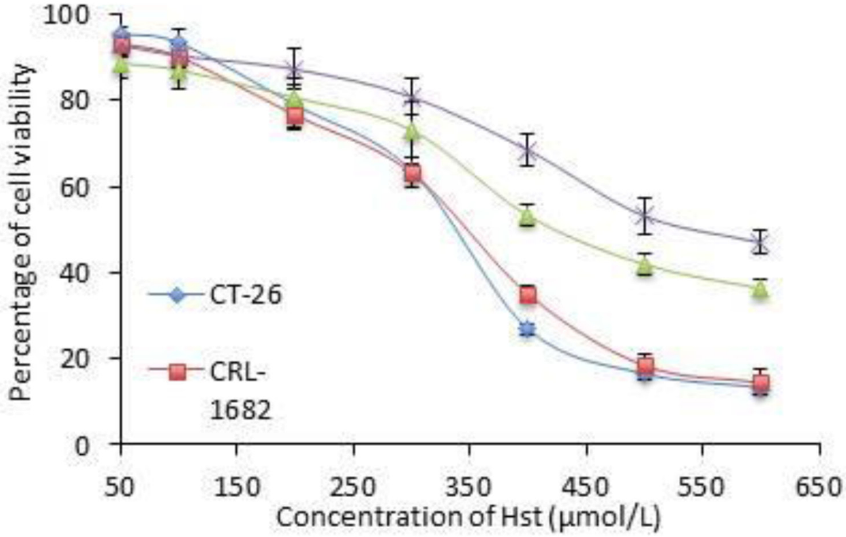

Supplementary Figure 4: Cell viability analysis of hesperetin on different cancer cell lines.

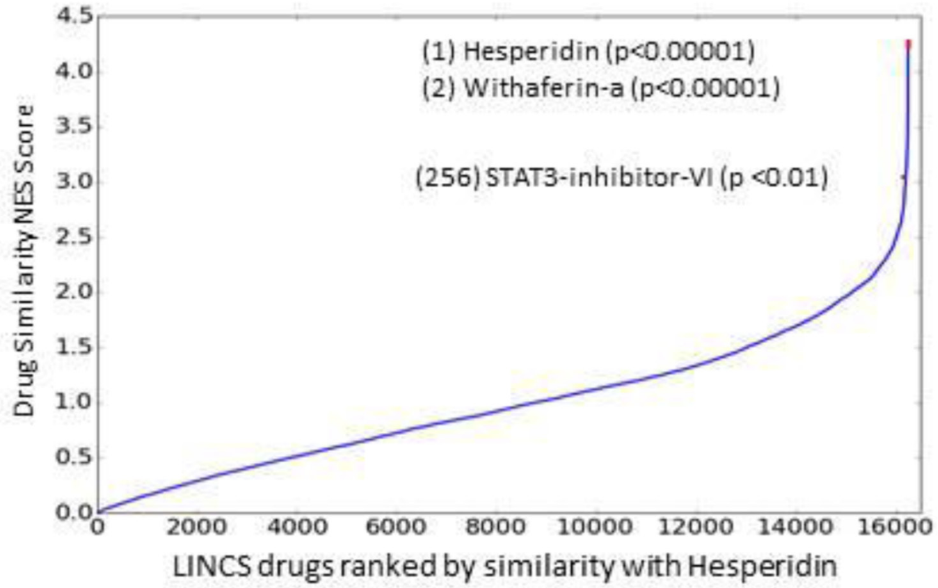

Supplementary Figure 5: LINCS drugs ranked by similarity with the glycoside Hst.

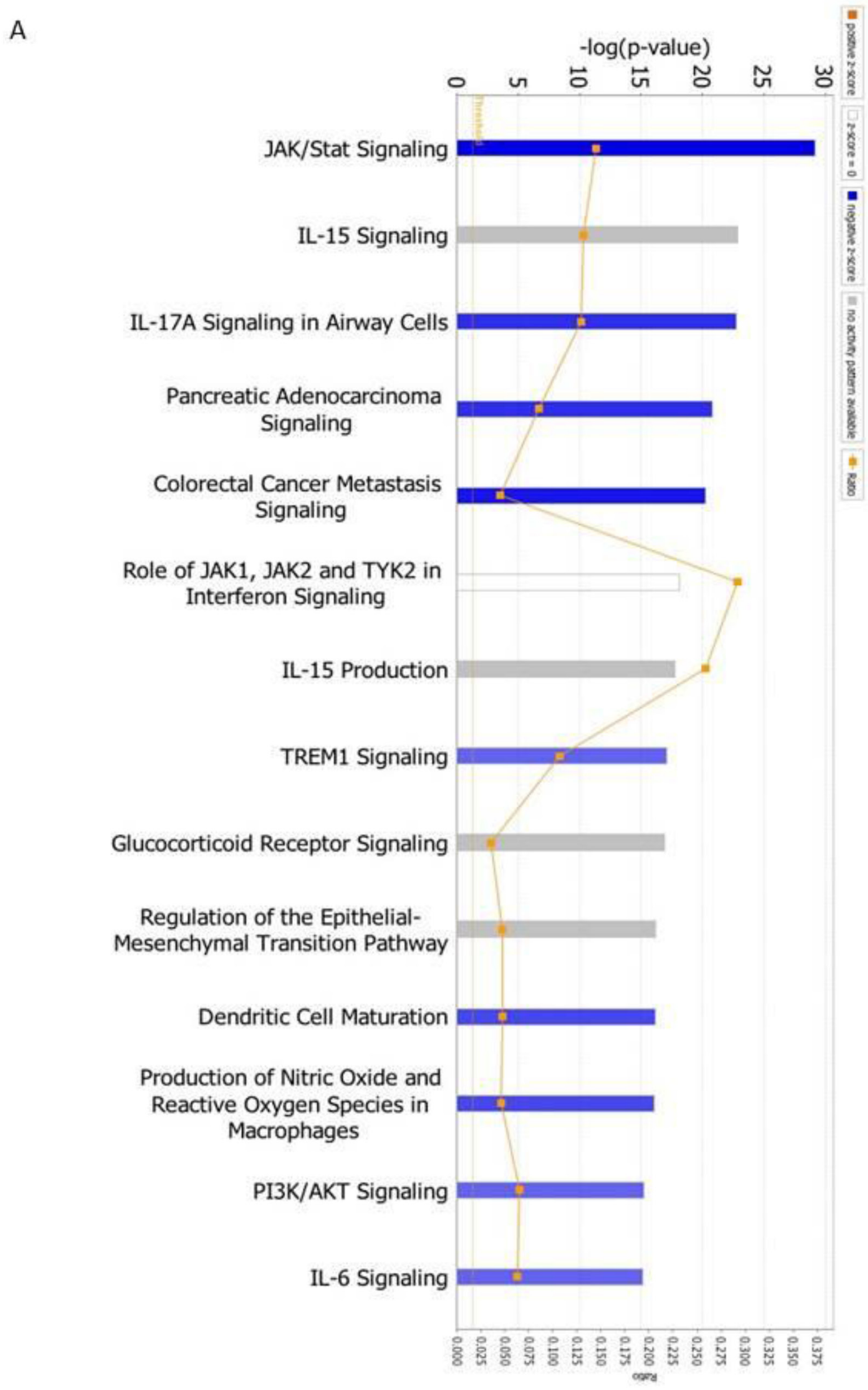

Supplementary Figure 6: Ingenuity pathway analysis of hesperidin. Gene expression data was extract from Lincscloud database. **(A)** Top enriched pathways of hesperidin down-regulates JAK/STAT pathway (p=6.24×10-30) and IL-6 Signaling (p=6.81×10-16).

(*Continued*)

B

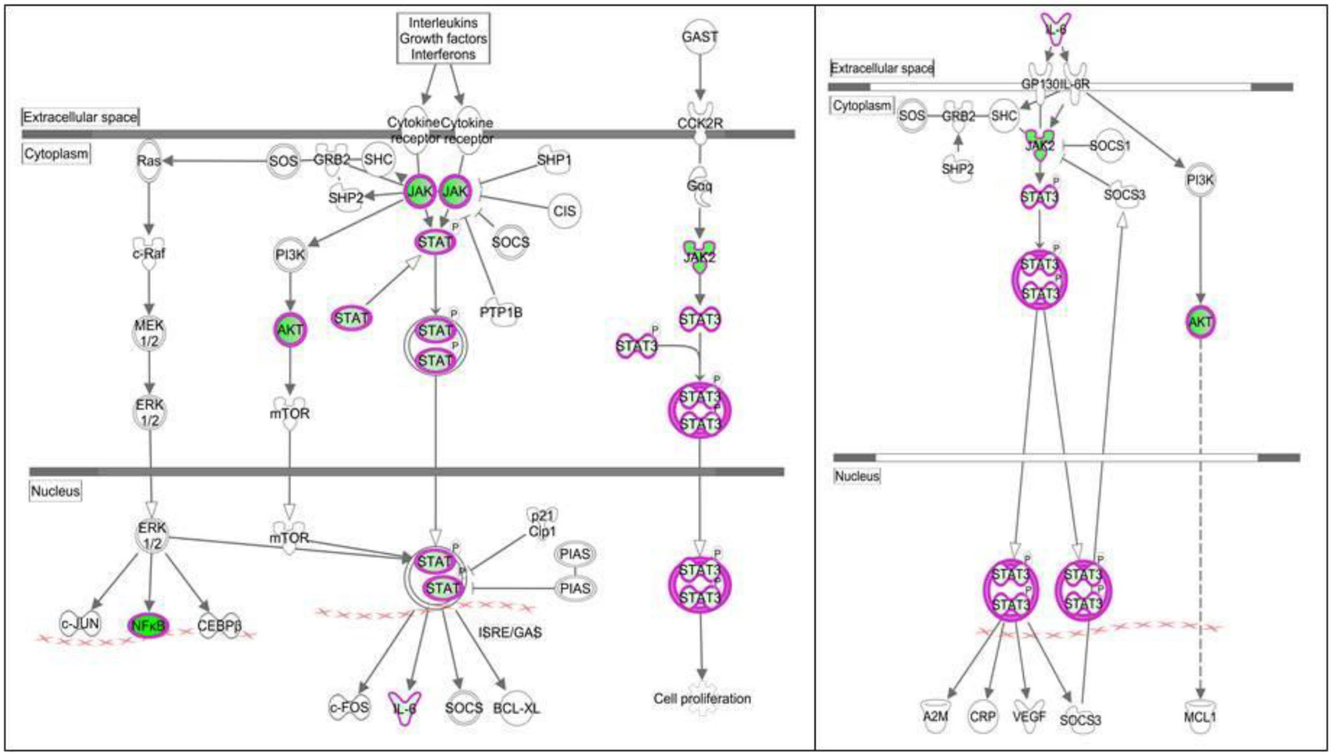

Supplementary Figure 6: (*Continued*) (B) Hesperidin regulations on JAK/STAT pathway and IL-6 induced STAT3 signaling.

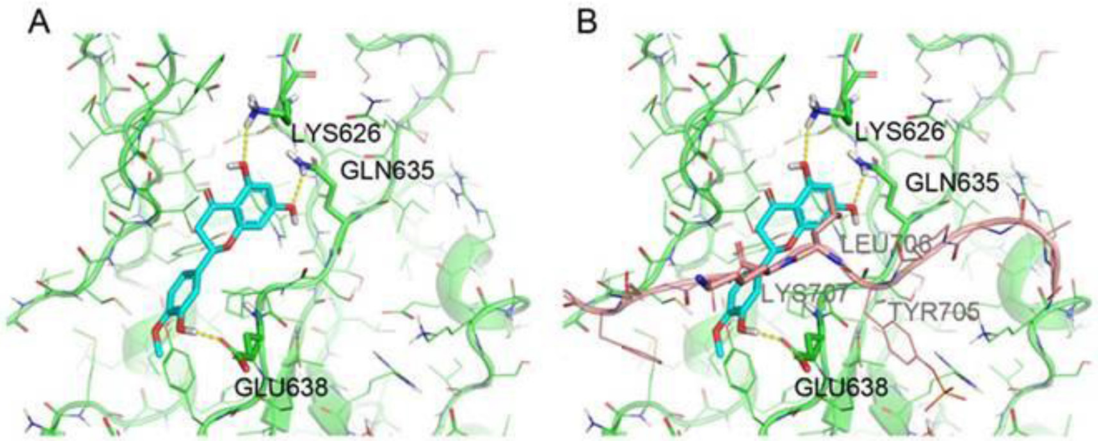

Supplementary Figure 7: (A) Binding pose of Hst with STAT3 generated by docking experiment (Glide). **(B)** Superimpose of phosphorylated peptide from crystal structure (PDB code 1BG1) and docking pose of Hst. STAT3 monomer used as receptor in docking experiments was shown in the cartoon model and colored in green, while the phosphorylated peptide from the other monomer in light pink. Hst and the residues formed hydrogen bond with it, including LYS626, GLN635 and GLU638 were shown in sticks model. The residues (LEU706, LYS707) overlapped with Hst were also shown in sticks. Other residues were shown in lines. The yellow dash lines represented hydrogen bonds between specific atoms. For clarity, all non-polar hydrogens were hided.

Supplementary Table 1: The structure and Glide docking score of the 196 compounds

See Supplementary File 1

Supplementary Table 2: The 24 compounds with >50% inhibition of CES2 enzyme activity in the primary screening

See Supplementary File 2
