# Supplementary material for: Two birds, one stone: hesperetin alleviates chemotherapy-induced diarrhea and potentiates tumor inhibition
Source: Oncotarget. 2018 Feb 23;9(46):27958–73. doi: 10.18632/oncotarget.24563 (PMC6021345; doi:10.18632/oncotarget.24563)
Supplement: Supplementary file 2 [file oncotarget-09-27958-s002.docx]

Supplementary Table 1: Structure and Glide docking score of the 196 compounds.

| Structure | ID | Glide Gscore | Structure | ID | Glide Gscore |
| --- | --- | --- | --- | --- | --- |
| 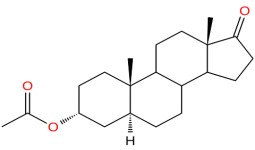 | 00107113 | -6.89 | 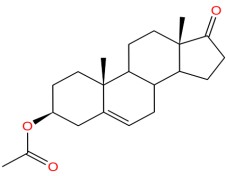 | 00270029 | -7.63 |
| 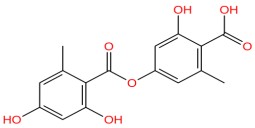 | 00200070 | -6.89 | 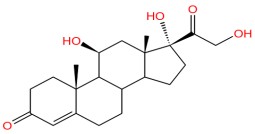 | 00300024 | -7.03 |
| 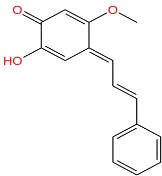 | 00200090 | -6.96 | 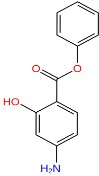 | 00305025 | -6.92 |
| 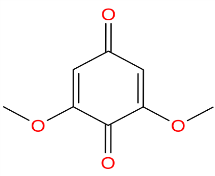 | 00200413 | -6.95 | 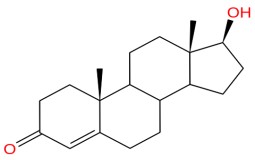 | 00307023 | -7.32 |
| 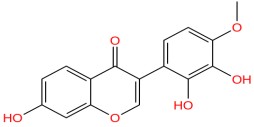 | 00200422 | -7.88 | 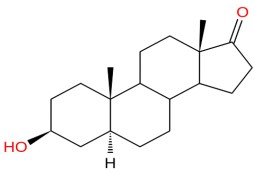 | 00310009 | -7.08 |
| 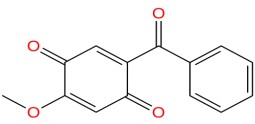 | 00200690 | -6.94 | 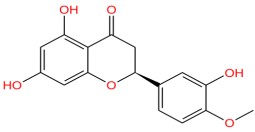 | 00310012 | -6.96 |
| 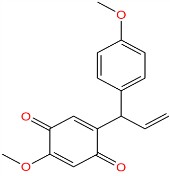 | 00201448 | -6.87 | 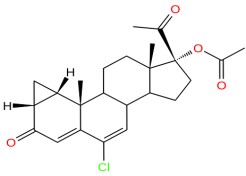 | 01500216 | -6.84 |
| 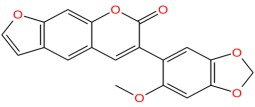 | 00201602 | -7.05 | 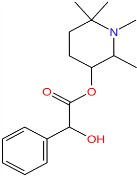 | 01500295 | -6.84 |

| 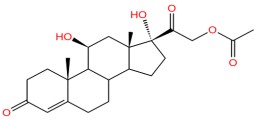 | 01500338 | -7.08 | 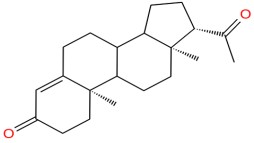 | 01500508 | -6.95 |
| --- | --- | --- | --- | --- | --- |
| 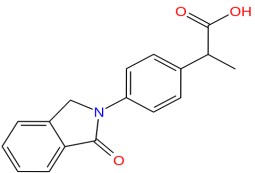 | 01500351 | -6.85 | 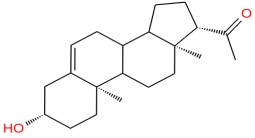 | 01500645 | -7.50 |
| 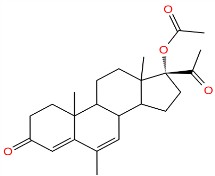 | 01500381 | -7.35 | 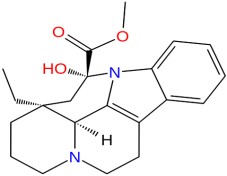 | 01500647 | -8.52 |
| 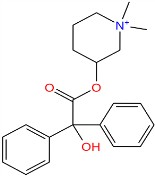 | 01500383 | -7.09 | 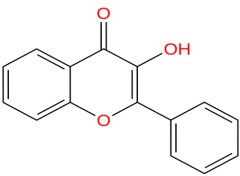 | 01501012 | -6.91 |
| 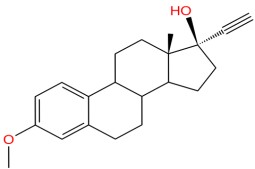 | 01500388 | -6.97 | 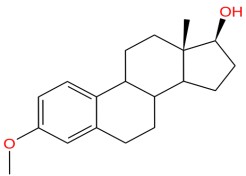 | 01501183 | -6.86 |
| 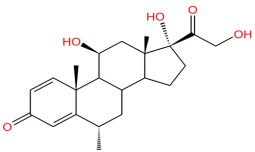 | 01500406 | -6.96 | 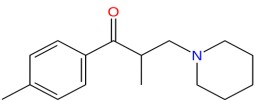 | 01501194 | -6.92 |
| 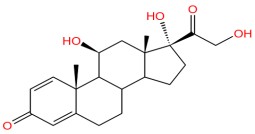 | 01500496 | -6.94 | 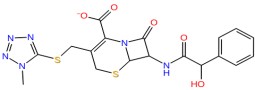 | 01502038 | -7.30 |
| 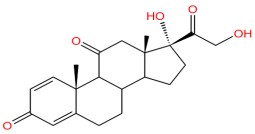 | 01500499 | -6.98 | 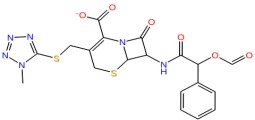 | 01502041 | -7.11 |

| 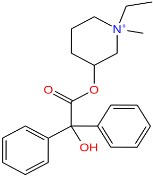 | 01503053 | -6.89 | 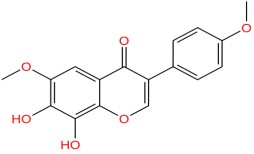 | 01505959 | -7.41 |
| --- | --- | --- | --- | --- | --- |
| 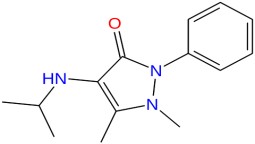 | 01503822 | -6.99 | 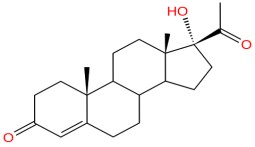 | 01701060 | -7.01 |
| 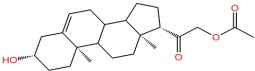 | 01505123 | -6.86 | 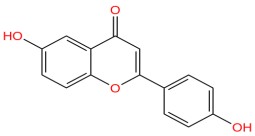 | 01500717 | -7.11 |
| 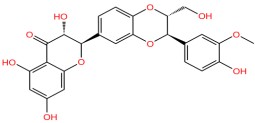 | 01505256 | -7.26 | 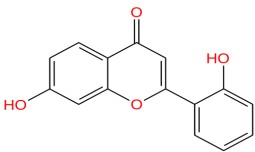 | 01500719 | -7.14 |
| 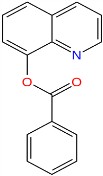 | 01505411 | -7.09 | 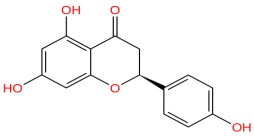 | 01500746 | -7.28 |
| 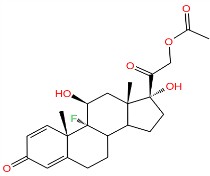 | 01505724 | -6.93 | 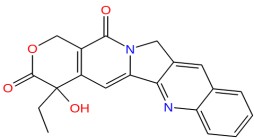 | 01502232 | -8.05 |
| 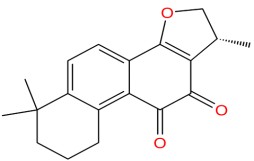 | 01505812 | -6.95 | 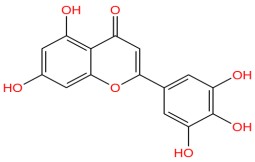 | 01504115 | -7.12 |
| 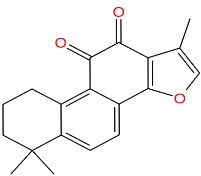 | 01505824 | -7.14 | 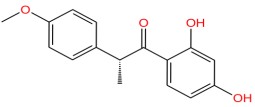 | 00100616 | -7.55 |

| 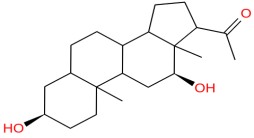 | 00100652 | -7.81 | 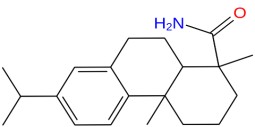 | 00307050 | -7.25 |
| --- | --- | --- | --- | --- | --- |
| 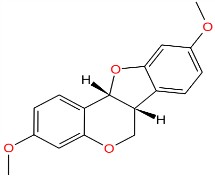 | 00100743 | -6.97 | 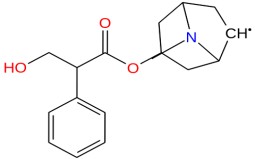 | 01500131 | -7.55 |
| 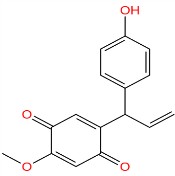 | 00200798 | -8.31 | 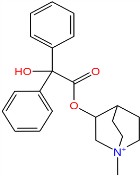 | 01500192 | -6.87 |
| 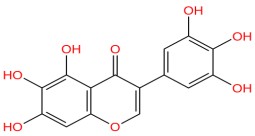 | 00201182 | -7.40 | 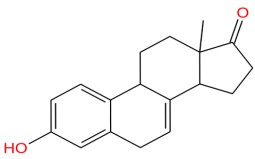 | 01500275 | -8.04 |
| 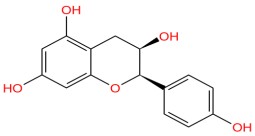 | 00202178 | -7.44 | 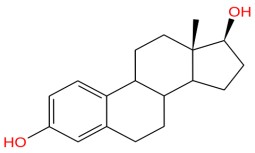 | 01500282 | -7.79 |
| 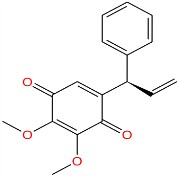 | 00240828 | -7.17 | 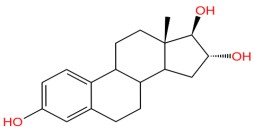 | 01500285 | -7.50 |
| 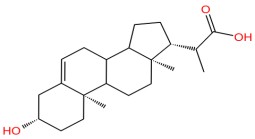 | 00270043 | -7.27 | 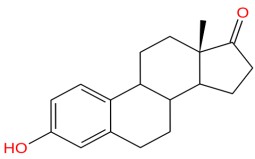 | 01500286 | -7.84 |
| 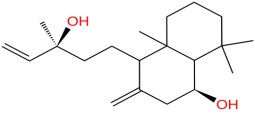 | 00300056 | -7.04 | 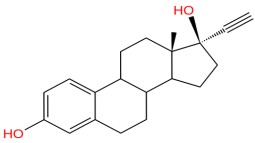 | 01500291 | -7.33 |

| 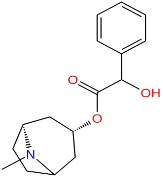 | 01500332 | -7.10 | 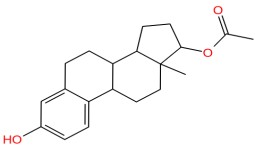 | 01501184 | -7.66 |
| --- | --- | --- | --- | --- | --- |
| 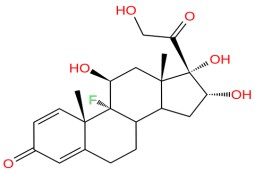 | 01500586 | -8.19 | 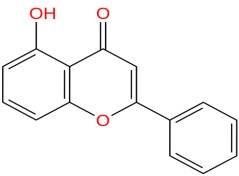 | 01501197 | -7.38 |
| 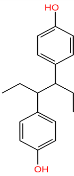 | 01500632 | -8.29 | 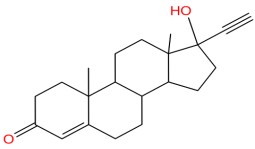 | 01503221 | -7.17 |
| 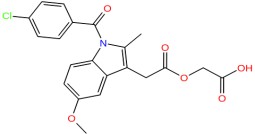 | 01500666 | -8.36 | 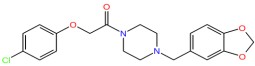 | 01503222 | -7.47 |
| 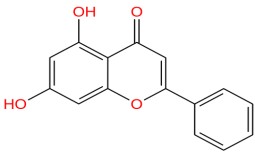 | 01500709 | -7.34 | 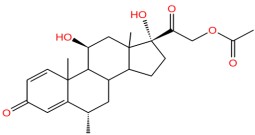 | 01503254 | -6.94 |
| 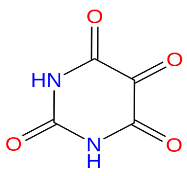 | 01500802 | -7.15 | 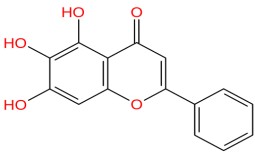 | 01504002 | -9.30 |
| 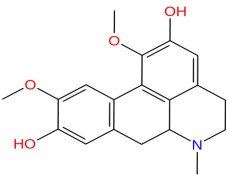 | 01500862 | -7.10 | 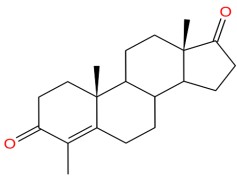 | 01504116 | -7.10 |
| 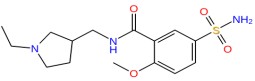 | 01501150 | -7.37 | 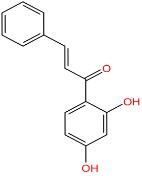 | 01505132 | -7.08 |

| 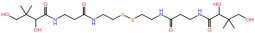 | 01505920 | -7.30 | 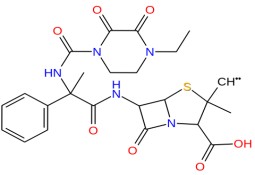 | JHU- 12498 | -6.91 |
| --- | --- | --- | --- | --- | --- |
| 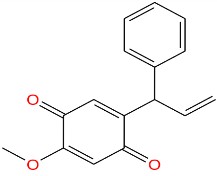 | 00201092 | -6.97 | 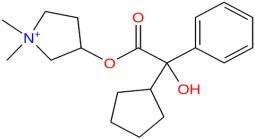 | JHU- 4657 | -6.96 |
| 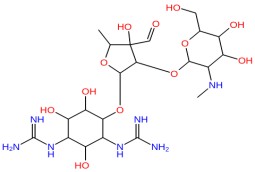 | JHU- 9449 | -7.60 | 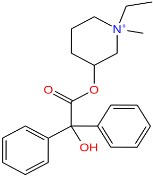 | JHU- 7920 | -7.24 |
| 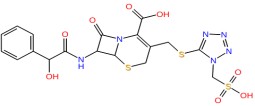 | JHU- 1889 | -7.26 | 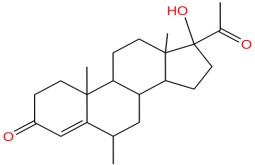 | JHU- 12397 | -6.98 |
| 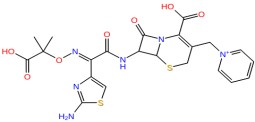 | JHU- 1914 | -7.61 | 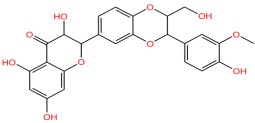 | JHU- 9113 | -7.47 |
| 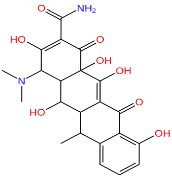 | JHU- 3350 | -7.35 | 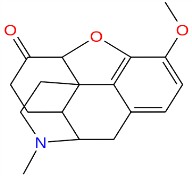 | JHU- 4885 | -8.57 |
| 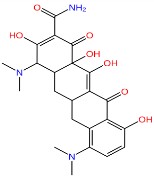 | JHU- 6505 | -7.08 | 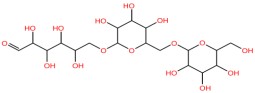 | JHU- 2953 | -7.05 |
| 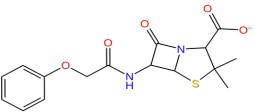 | JHU- 12471 | -8.60 | 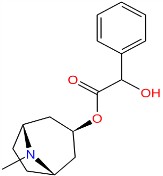 | JHU- 12331 | -7.54 |

| 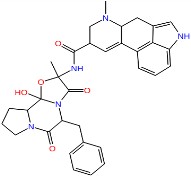 | JHU- 3685 | -7.63 | 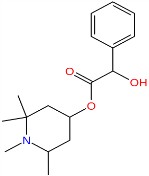 | JHU- 3912 | -7.19 |
| --- | --- | --- | --- | --- | --- |
| 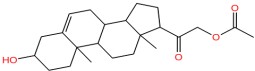 | JHU- 16 | -7.04 | 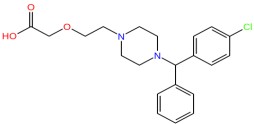 | JHU- 1984 | -7.32 |
|  | JHU- 8271 | -6.89 |  | JHU- 12836 | -7.36 |
|  | JHU- 8274 | -6.93 |  | JHU- 12844 | -7.39 |
|  | JHU- 8882 | -7.15 |  | JHU- 12165 | -6.87 |
|  | JHU- 9113 | -7.29 |  | JHU- 5976 | -6.92 |
|  | JHU- 2694 | -7.05 |  | JHU- 4068 | -7.22 |
|  | JHU- 2117 | -6.93 |  | JHU- 3738 | -8.26 |

|  | JHU- 7072 | -7.13 |  | JHU- 2660 | -6.98 |
| --- | --- | --- | --- | --- | --- |
|  | JHU- 1136 | -6.93 |  | JHU- 636 | -6.83 |
|  | JHU- 6136 | -7.30 |  | JHU- 3629 | -8.13 |
|  | JHU- 6074 | -7.09 |  | JHU- 3849 | -7.22 |
|  | JHU- 7302 | -6.89 |  | JHU- 8285 | -7.59 |
|  | JHU- 9409 | -7.24 |  | JHU- 12372 | -6.86 |
|  | JHU- 10444 | -7.30 |  | JHU- 12321 | -7.61 |
|  | JHU- 8250 | -7.89 |  | A-236 | -7.97 |

|  | A 0232 | -7.22 |  | I-146 | -7.06 |
| --- | --- | --- | --- | --- | --- |
|  | C-141 | -6.98 |  | I-122 | -8.00 |
|  | PZ0104 | -7.35 |  | D 8941 | -7.81 |
|  | D 5439 | -7.13 |  | B9061 | -7.22 |
|  | E 3132 | -7.41 |  | S-154 | -7.18 |
|  | F 6020 | -6.85 |  | T 4512 | -7.09 |
|  | H 0126 | -6.88 |  | A 0257 | -7.57 |
|  | L 2167 | -7.26 |  | A 4393 | -7.59 |

|  | I0404 | -7.35 |  | E-101 | -7.72 |
| --- | --- | --- | --- | --- | --- |
|  | A 9345 | -7.11 |  | F 6145 | -6.99 |
|  | K3519 | -7.18 |  | E 8875 | -7.79 |
|  | D 1916 | -7.69 |  | E 9750 | -7.83 |
|  | D 7802 | -7.60 |  | G 6649 | -8.04 |
|  | D-003 | -7.85 |  | E 7881 | -8.94 |
|  | D-027 | -7.14 |  | H 9002 | -7.55 |
|  | D-029 | -7.44 |  | K 1136 | -7.52 |

|  | I 3766 | -6.89 |  | R 5010 | -6.90 |
| --- | --- | --- | --- | --- | --- |
|  | L 4376 | -6.92 |  | S 8567 | -6.85 |
|  | M 6383 | -7.31 |  | T-104 | -6.99 |
|  | N-151 | -7.47 |  | 08E08 | -7.11 |
|  | P 0453 | -7.10 |  | 01C05 | -7.06 |
|  | P-215 | -6.85 |  | 01G03 | -7.29 |
|  | D5446 | -8.83 |  | 02C02 | -7.46 |
|  | T2952 | -7.16 |  | 03H04 | -6.94 |

|  | 04D02 | -7.10 |  | 09B03 | -6.92 |
| --- | --- | --- | --- | --- | --- |
|  | 06G07 | -7.49 |  | 10C04 | -6.89 |
|  | 07B07 | -6.92 |  | 10C07 | -6.96 |
| O O  +  N  O N CH3  H3  OH | 08A10 | -7.78 |  | 10H05 | -7.00 |
|  | 08D11 | -6.88 |  | 11B08 | -7.67 |
|  | 08F06 | -7.92 |  | 11G11 | -7.13 |
|  | 08H08 | -7.48 |  | 11H03 | -7.74 |
|  | 09A11 | -8.29 |  | 11H09 | -7.55 |

HO C N

|  | 12F03 | -7.29 |
| --- | --- | --- |
|  | 13B02 | -6.91 |
|  | 14F07 | -7.39 |
|  | 14F09 | -7.11 |
